# IL-17B alleviates the pathogenesis of systemic lupus erythematosus by inhibiting FASN-mediated differentiation of B cells

**DOI:** 10.1172/jci.insight.181906

**Published:** 2024-08-08

**Authors:** Yucai Xiao, Yuxin Hu, Yangzhe Gao, Lin Wang, Lili Zhang, Qun Ma, Zhaochen Ning, Lu Yu, Haochen Li, Jiakun Liu, Junyu Wang, Yonghong Yang, Huabao Xiong, Guanjun Dong

**Affiliations:** 1 Cheeloo College of Medicine, Shandong University, Jinan, Shandong, China.; 2 Institute of Immunology and Molecular Medicine, and; 3 Jining Key Laboratory of Immunology, Jining Medical University, Shandong, China.; 4 Department of Rheumatology and; 5 Medical Research Center, Affiliated Hospital of Jining Medical University, Jining, Shandong, China.

**Keywords:** Autoimmunity, Autoimmune diseases, Lupus

## Abstract

The interleukin 17 (IL-17) family of cytokines has emerged as a critical player in autoimmune disease, including systemic lupus erythematosus (SLE). However, the role of IL-17B, a poorly understood cytokine, in the pathogenesis of SLE is still not known. In this study, we investigated the role of IL-17B in the activation and differentiation of B cells, and the pathogenesis of SLE. Intriguingly, IL-17B deficiency aggravated disease in lupus-prone mice and promoted the activation of B cells and the differentiation of germinal center B cells and plasma cells, while recombinant mouse IL-17B (rmIL-17B) significantly alleviated disease in lupus-prone mice. Mechanistically, rmIL-17B inhibited the activation of the Toll-like receptor and interferon pathways in B cells by downregulating fatty acid synthase–mediated (FASN-mediated) lipid metabolism. Loss of FASN significantly alleviated the disease in lupus-prone mice and inhibited the activation and differentiation of B cells. In addition, B cells had greater FASN expression and lower IL-17RB levels in patients with SLE than in healthy controls. Our study describes the role of IL-17B in regulating B cell activation and differentiation, and alleviating the onset of SLE. These findings will lay a theoretical foundation for further understanding of the pathogenesis of SLE.

## Abstract 2

IL-17B alleviates the pathogenesis of SLE.

## Graphical abstract

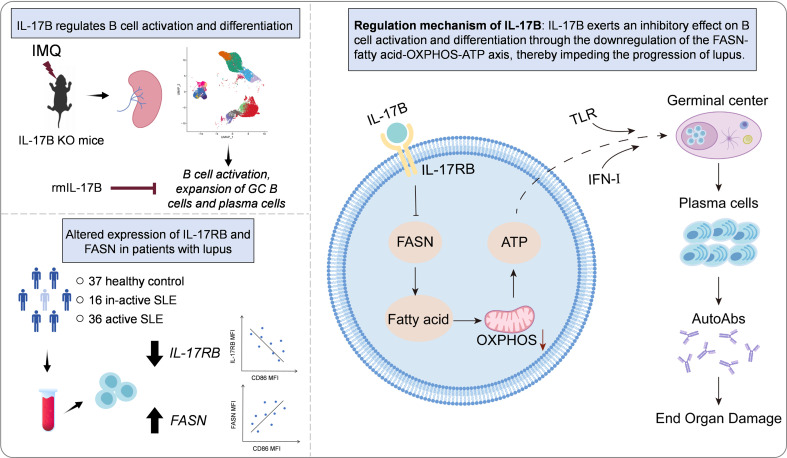

## Introduction

Systemic lupus erythematosus (SLE) is a chronic autoimmune disease in which multiple systems in the body are affected, and effective treatment options are lacking (1). Although the pathogenesis of SLE involves various factors, such as genetics, environment, and immunity, numerous studies have shown that B cells play a critical role in the pathogenesis of SLE (2). Aberrant activation of B cells leads to unwanted differentiation of B cells into plasma cells, followed by the production of a large number of pathogenic autoantibodies; these changes lead to systemic organ and tissue damage (3). The germinal center (GC) serves as a crucial locus for the differentiation and maturation of B cells, where B cells rapidly amplify, undergo a high frequency of somatic maturation and antibody class switching, and differentiate into high-affinity memory B cells and long-lived plasma cells. Although several studies have confirmed that Toll-like receptors (TLRs) and interferons (IFNs) can regulate the differentiation of GC B cells and contribute to the pathogenesis of SLE (4–6), the precise regulatory mechanisms underlying the abnormal activation and differentiation of GC B cells need to be fully elucidated.

Metabolic reprogramming regulates the activation and differentiation of immune cells and influences immune-related diseases (7, 8). During activation and differentiation, immune cells such as B cells require considerable energy and need to rapidly synthesize biological components using lipids, amino acids, and nucleic acids. Our findings, along with those of other researchers, have shown that metabolism participates in regulating the activation and differentiation of B cells (9–11). However, few studies have investigated the metabolic status of GC B cells, which undergo rapid proliferation and differentiation. Interestingly, one study showed that abnormal lipid metabolism has a strong regulatory effect on the differentiation of GC B cells (12). GC B cells exhibit high levels of fatty acid oxidation, but low levels of glycolysis, during proliferation and differentiation; thus, they use fatty acids, particularly long-chain fatty acids, as their primary fuel source for mitochondrial oxidative phosphorylation (OXPHOS). Therefore, GC B cells have a high capacity for mitochondrial respiration to meet their energy requirements and support their proliferation and differentiation (12). Notably, fatty acid synthase (FASN) serves as a rate-limiting enzyme in the fatty acid synthesis pathway. It facilitates the conversion of acetyl-CoA and malonyl-CoA into long-chain saturated fatty acids such as palmitic acid or octadecanoic acid in the presence of NADPH. The synthesized saturated fatty acids can be further transformed into monounsaturated fatty acids or incorporated into different types of lipids, such as various phospholipids, diacylglycerols, and triacylglycerols (8). However, the precise molecular mechanism underlying abnormal lipid metabolism in GC B cells and the mechanism by which lipid metabolism regulates the differentiation of GC B cells need to be elucidated.

Interleukin 17B (IL-17B) is 1 of the 6 members of the IL-17 family; the other members include IL-17A, IL-17C, IL-17D, IL-17E, and IL-17F. Some studies have shown that cytokines in the IL-17 family play a key role in host defense responses on skin and mucosal surfaces (13–15). Specifically, IL-17A, IL-17F, and IL-17C participate in neutrophil-mediated responses to protect the body against bacterial and fungal infections (15, 16). IL-17E contributes to antiparasitic immunity due to its association with allergies and atopic dermatitis (17). In contrast, the functional role of IL-17B remains mostly unknown. Several studies have reported that IL-17B can promote inflammatory arthritis in mice (18) and participate in the development of tumors (19, 20), while another study showed that IL-17B can alleviate dextran sulfate sodium–induced colitis (21). There are still contradictions in the immune regulation of IL-17B. Notably, the role of IL-17B in the pathogenesis of SLE has not been reported.

In this study, we found that IL-17B plays a negative role in the pathogenesis of SLE by inhibiting the differentiation of GC B cells and plasma cells. Upon investigating the mechanism of action, we found that IL-17B suppressed B cell activation and differentiation by modulating the FASN/mitochondrial OXPHOS/ATP axis, thus mitigating the pathogenesis of SLE. In addition, its receptor (IL-17RB) was significantly downregulated, and FASN was significantly overexpressed in the B cells of patients with SLE compared with the corresponding levels in the B cells of healthy individuals. Our study provides a foundation for understanding the etiology of SLE and for exploring the role of IL-17B in the pathogenesis of SLE.

## Results

### IL-17B plays a protective role in the pathogenesis of lupus in mice.

**Figure 1:**
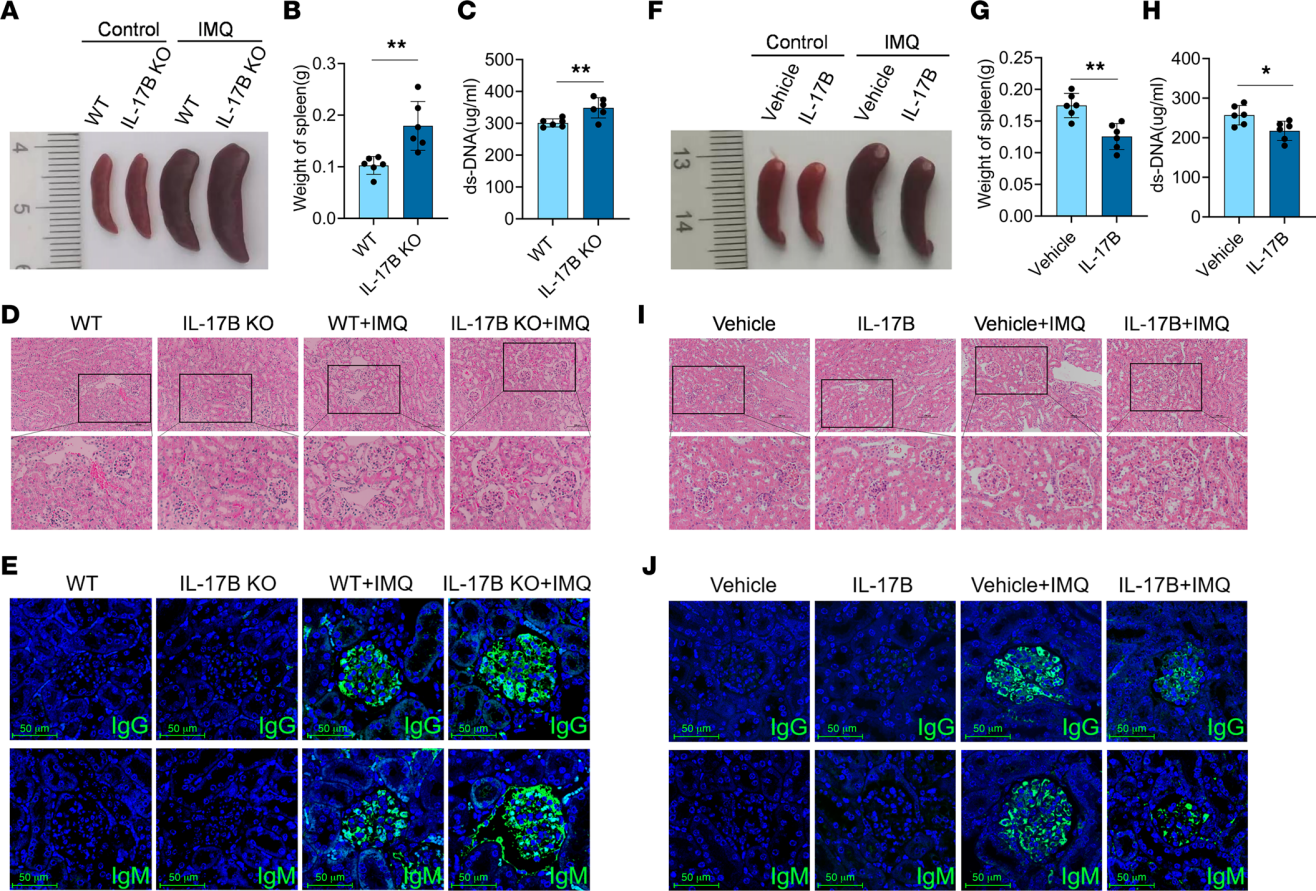
IL-17B attenuates IMQ-induced disease progression in mice with lupus. (**A**) Splenic images, (**B**) spleen weights, (**C**) serum levels of anti-dsDNA antibodies, (**D**) H&E staining of kidney, and (**E**) renal IgG and IgM deposition in WT or *IL-17B*–KO mice treated with IMQ. (**F**) Splenic images, (**G**) spleen weights, (**H**) serum levels of anti-dsDNA antibodies, (**I**) H&E staining of kidney, and (**J**) renal IgG and IgM deposition in IMQ-induced WT mice treated with vehicle or rmIL-17B. Scale bars: 100 μm. The data are shown as the mean ± SEM and are representative of 3 independent experiments (*n* = 6 mice/group). *P < 0.05, **P < 0.01 by 2-tailed Student’s *t* test.

To investigate the role of IL-17B in the pathogenesis of lupus, IL-17B gene–KO mice were generated and used to construct a lupus model using imiquimod (IMQ). Intriguingly, we found that the spleen was enlarged to a greater extent in IMQ-treated *IL-17B*–KO mice than in wild-type (WT) mice (Figure 1A). Compared with WT mice, IMQ-treated *IL-17B*–KO mice had significantly greater spleen weights (Figure 1B) and greater levels of anti–double-stranded DNA (anti-dsDNA) antibodies (Figure 1C). By analyzing H&E-stained tissues, we found that IL-17B deficiency exacerbated kidney injury in IMQ-treated mice, as characterized by substantial enlargement of the glomerulus and segmental mesangial matrix hyperplasia (Figure 1D and Supplemental Figure 1A; supplemental material available online with this article; https://doi.org/10.1172/jci.insight.181906DS1). Additionally, IMQ-treated *IL-17B–*KO mice had significantly greater amounts of IgG and IgM deposited in the glomeruli than did WT mice (Figure 1E and Supplemental Figure 1, B and C). These findings suggested that IL-17B deficiency exacerbates disease progression in a mouse model of lupus.

We next investigated whether recombinant mouse IL-17B (rmIL-17B) could improve the condition of lupus model mice. Compared with vehicle-treated IMQ mice, rmIL-17B–treated IMQ mice exhibited greater reductions in splenomegaly severity (Figure 1F), spleen weights (Figure 1G), serum anti-dsDNA antibody levels (Figure 1H), kidney injury (Figure 1I and Supplemental Figure 1D), and the deposition of IgG and IgM in the glomeruli (Figure 1J and Supplemental Figure 1, E and F). Moreover, rmIL-17B effectively improved the condition of MRL/*lpr* lupus model mice (Supplemental Figure 2, A–G), indicating that IL-17B plays a protective role in the pathogenesis of lupus-prone mice.

### IL-17B significantly inhibited the activation and differentiation of B cells in vivo.

**Figure 2:**
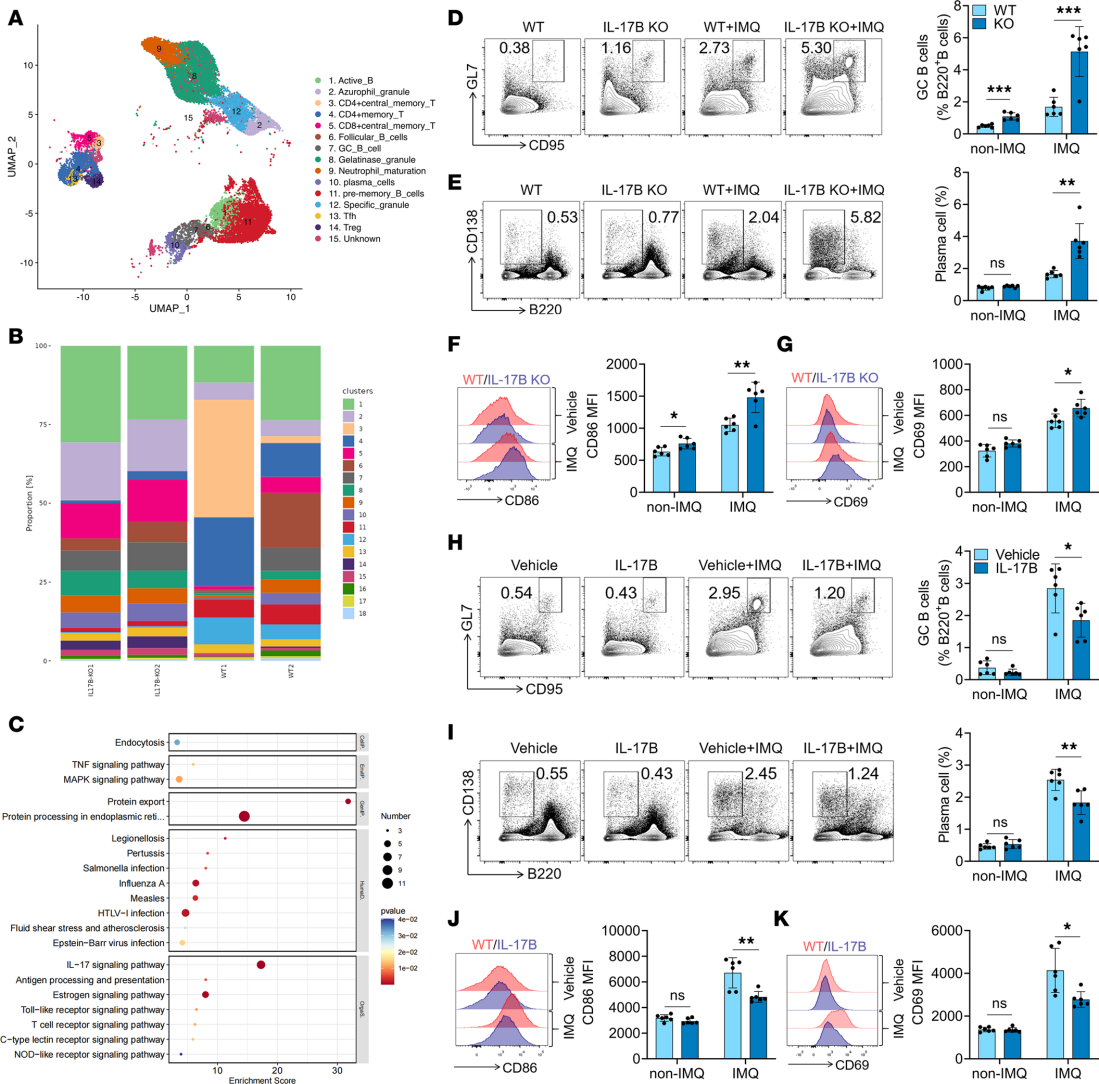
IL-17B represses B cell activation and differentiation in vivo. (**A**) Uniform Manifold Approximation and Projection plot of spleen cells from IMQ-treated WT (*n* = 2) and *IL-17B*–KO mice (*n* = 2). (**B**) Proportion of cell types in the splenocytes of IMQ-treated WT (*n* = 2) or *IL-17B*–KO mice (*n* = 2). (**C**) KEGG analysis of splenic GC B cells from IMQ-treated WT and *IL-17B*–KO mice. Representative flow cytometry images and statistical analysis of the percentages of (**D**) splenic GC B cells (B220^+^GL-7^+^CD95^+^), (**E**) plasma cells (CD138^+^B220^–^), and the expression of (**F**) CD86 and (**G**) CD69 on B220^+^ B cells in IMQ-treated WT or *IL-17B*–KO mice. The percentages of (**H**) spleen GC B cells, (**I**) plasma cells, and the expression of (**J**) CD86 and (**K**) CD69 on B220^+^ B cells in IMQ-induced WT mice treated with vehicle or rmIL-17B. The data are shown as the mean ± SEM and are representative of 3 independent experiments. *P < 0.05, **P < 0.01, ***P < 0.001 by 2-tailed Student’s *t* test. NS, *P* > 0.05.

To elucidate the mechanism by which IL-17B alleviates the pathogenesis of lupus, single-cell RNA sequencing (scRNA-seq) was performed using splenocytes from IMQ-treated WT and *IL-17B–*KO mice. Splenic cell populations were clustered based on the cell transcriptional profile. The marker genes that defined each cell cluster are presented in Supplemental Figure 3A. For example, GC B cells specifically expressed *Tubb5*, *Pclaf*, *Top2a*, *Mki67*, and *Myc*, and plasma cells specifically expressed *Prdm1*, *Xbp1*, *Sdc1*, and *Trp53inp1*. The identity of the cells in each cluster was determined by evaluating differentially expressed genes within the clusters (Figure 2A and Supplemental Figure 3B). We analyzed the proportions of each cell cluster to determine differences in the composition of B cell and T cell subsets between *IL-17B*–KO and WT mice. Notably, compared with WT mice, *IL-17B*–KO mice exhibited significantly greater proportions of GC B cells and plasma cells among B cells (Figure 2B). Supplemental Figure 3C shows the top 20 genes whose expression was upregulated or downregulated in GC B cells from IMQ-treated *IL-17B*–KO mice compared with those from IMQ-treated WT mice. Kyoto Encyclopedia of Genes and Genomes (KEGG) pathway analysis revealed that IMQ-treated *IL-17B*–KO mice exhibited significant enrichment of the TLR and MAPK signaling pathways, which are related to the activation and differentiation of B cells (Figure 2C). Gene Ontology (GO) analysis revealed significant enrichment of the IFN-I response in *IL-17B*–KO mice induced by IMQ (Supplemental Figure 3D).

Next, we examined the effect of IL-17B on the activation and differentiation of B cells in IMQ-treated mice. As shown in Figure 2, D–G, IL-17B deficiency led to a marked increase in the proportion of GC B cells and plasma cells in the spleens of IMQ-treated mice, as well as in the expression of CD86 and CD69 on B cells. Similar results were also found in mesenteric lymph nodes (mLNs) from these mice (Supplemental Figure 4, A–C). In contrast, administration of rmIL-17B significantly reduced the proportions of GC B cells (Figure 2H) and plasma cells (Figure 2I) in the spleens of IMQ-treated mice and decreased CD86 and CD69 expression on B cells (Figure 2, J and K). Similar results were also found for the mLNs from these mice (Supplemental Figure 5, A–C). These results suggest that IL-17B can indeed regulate the activation and differentiation of B cells.

Follicular helper T (Tfh) cells play a critical role in the differentiation of GC B cells. scRNA-seq revealed that IL-17B deficiency led to a marked increase in the proportion of Tfh and memory CD4^+^ T cells in the spleens of IMQ mice (Figure 2B). Flow cytometry analysis revealed that, compared with WT mice, IMQ-treated *IL-17B*–KO mice exhibited significantly greater proportions of Tfh and memory CD4^+^ T cells and increased CD69 expression on CD4^+^ T cells in the spleen (Supplemental Figure 4, D–F) and mLNs (Supplemental Figure 4, G and H). As expected, administration of rmIL-17B considerably reduced the proportion of Tfh and memory CD4^+^ T cells and decreased CD69 expression on CD4^+^ T cells in the spleen (Supplemental Figure 5, D–F) and mLNs (Supplemental Figure 5, G and H) of IMQ-treated mice.

Furthermore, we examined whether IL-17B can affect the GC response in sheep red blood cell–immunized (SRBC-immunized) mice. As expected, treatment with rmIL-17B could significantly downregulate the proportions of GC B cells, plasma cells, and Tfh cells in the SRBC-immunized mice (Supplemental Figure 6, A–D), suggesting that IL-17B has the capacity to suppress the GC response elicited by exogenous antigens. All these results suggest that IL-17B exerts a beneficial effect on lupus by inhibiting the activation and differentiation of B cells and T cells.

### IL-17B inhibited the activation of the TLR and IFN-I signaling pathways in B cells in vitro.

**Figure 3:**
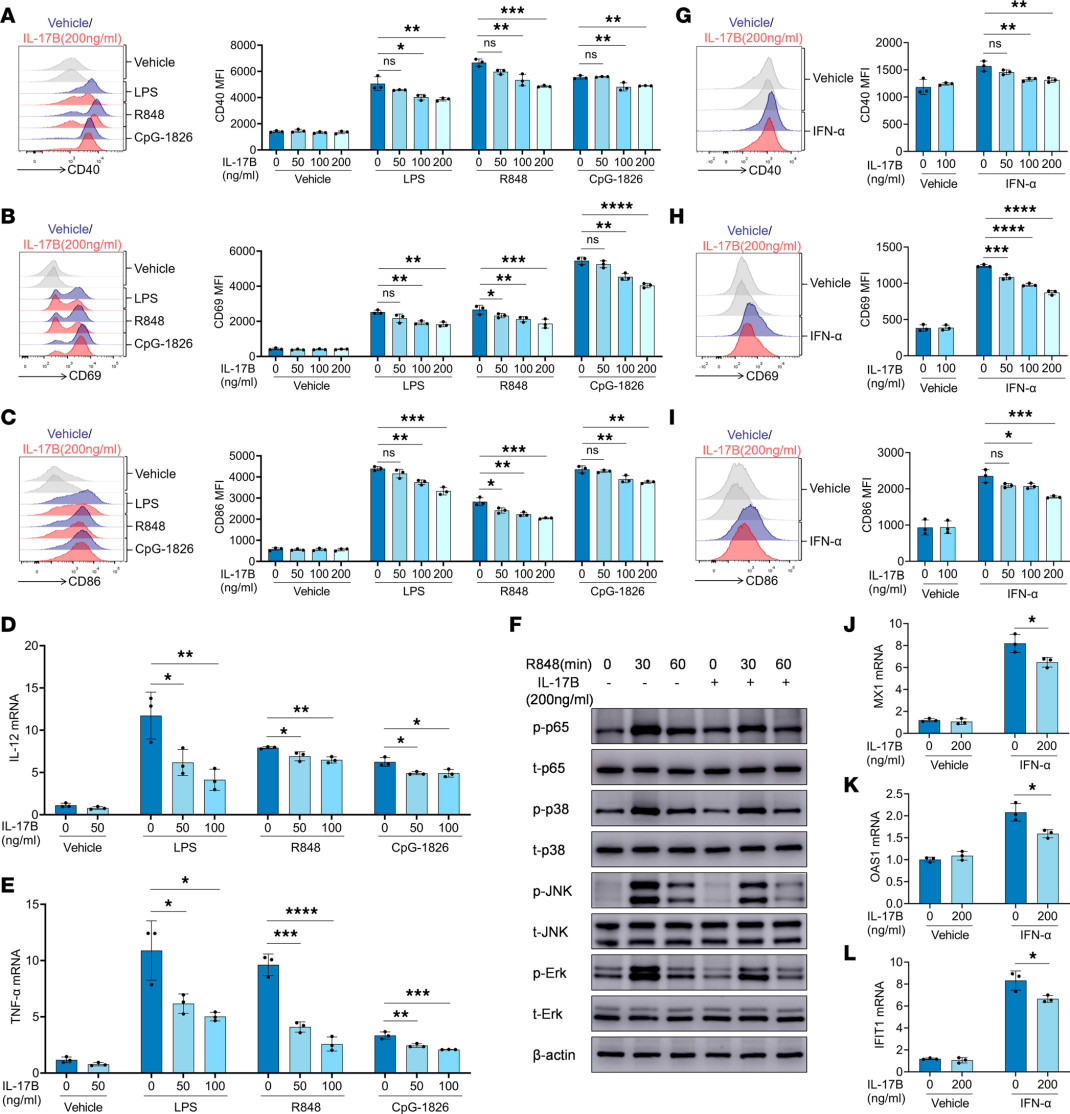
IL-17B inhibits B cell activation induced by TLRs and IFN-I pathways in vitro. WT mice spleen B cells were pretreated with rmIL-17B for 12 hours and stimulated with LPS (100 ng/mL), R848 (1 μg/mL), and CpG-1826 (1 μM). (**A**) CD40, (**B**) CD69, and (**C**) CD86 expression at 24 hours. (**D**) IL-12 and (**E**) TNF-α mRNA levels at 6 hours. (**F**) Total (t-) and phosphorylated (p-) p65, p38, JNK, and Erk at 30 or 60 minutes after R848 stimulation. (**G**) CD40, (**H**) CD69, and (**I**) CD86 expression at 24 hours, and (**J**) MX1, (**K**) OAS1, and (**L**) IFIT1 mRNA levels at 6 hours in WT mice spleen B cells pretreated with rmIL-17B for 12 hours and stimulated with IFN-α (1000 U/mL). The data are shown as the mean ± SEM and are representative of 3 independent experiments. *P < 0.05, **P < 0.01, ***P < 0.001, ****P < 0.0001 by 1-way ANOVA with Tukey’s multiple-comparison test (**A**–**E** and **G**–**I**) or 2-way ANOVA with Bonferroni’s mutiple-comparison test (**J**–**L**). NS, *P* > 0.05.

Considering that the TLR and IFN-I signaling pathways participate in regulating the activation and differentiation of B cells (4), we next investigated the effect of IL-17B on the activation of the TLR and IFN-I signaling pathways in B cells. Murine naive B cells were treated with rmIL-17B for 12 hours, followed by stimulation with the TLR4 agonist LPS, TLR7 agonist R848, and TLR9 agonist CpG-1826. Notably, treatment with rmIL-17B markedly reversed the TLR ligand–induced expression of CD40 (Figure 3A), CD69 (Figure 3B), and CD86 (Figure 3C), as well as the expression of IL-12 (Figure 3D) and TNF-α (Figure 3E). In addition, treatment with rmIL-17B considerably decreased the R848-induced phosphorylation of p65, p38, JNK, and Erk (Figure 3F and Supplemental Figure 7, A–D). The effect of IL-17B on the activation of IFN-I signaling pathways in B cells was also investigated. Murine naive B cells were treated with rmIL-17B for 12 hours, followed by stimulation with IFN-α. As shown in Figure 3, G–I, treatment with rmIL-17B significantly reversed the IFN-α–induced increase in the expression of CD40, CD69, and CD86. Moreover, treatment with rmIL-17B inhibited the IFN-α–induced expression of MX1, OAS1, and IFIT1 (Figure 3, J–L) in B cells. Together, these findings suggest that IL-17B can inhibit and modulate the activation of the TLR and IFN-I signaling pathways in B cells in vitro.

### IL-17B inhibited the activation of TLR and IFN-I pathways by downregulating FASN expression.

**Figure 4:**
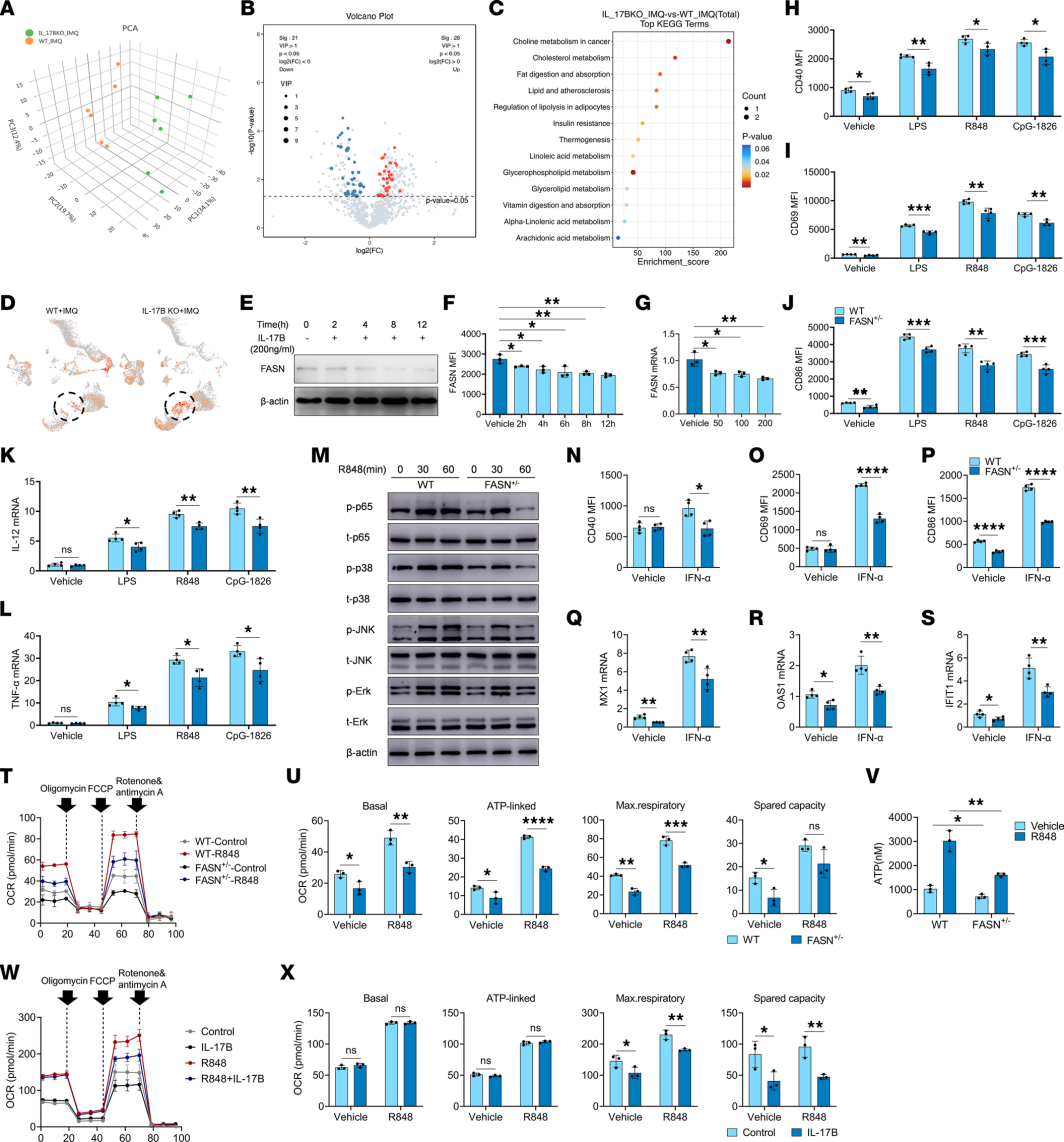
IL-17B suppresses the activation of B cells by attenuating the FASN/OXPHOS/ATP axis. (**A**) PCA plot, (**B**) volcano plot, and (**C**) KEGG analysis of metabolites generated from serum lipid profiles of IMQ-treated WT (*n* = 6) and *IL-17B*–KO mice (*n* = 6). (**D**) The FASN intensity on each cell from IMQ-treated WT mice (*n* = 2) and *IL-17B*–KO mice (*n* = 2) was expressed as *z* score–normalized expression. Dashed-line circles indicate GC B cells. (**E** and **F**) Western blot and flow cytometry analysis of FASN expression in B cells after different time periods of rmIL-17B treatment. (**G**) Quantitative PCR analysis was performed to assess the expression of FASN in B cells following treatment with varying concentrations of rmIL-17B. (**H**–**M**) B cells, isolated from the spleen of WT mice or *FASN^+/–^* mice, were stimulated with LPS, R848, and CpG-1826. Expression of (**H**) CD40, (**I**) CD69, and (**J**) CD86 at 24 hours. mRNA levels of (**K**) IL-12 and (**L**) TNF-α at 6 hours. (**M**) Phosphorylation of p65, p38, JNK, and Erk. (**N**–**S**) B cells, isolated from the spleen of WT mice or *FASN^+/–^* mice, were stimulated with IFN-α. (**N**) CD40, (**O**) CD69, and (**P**) CD86 expression at 24 hours. (**Q**) MX1, (**R**) OAS1, and (**S**) IFIT1 mRNA levels at 6 hours. (**T**–**V**) B cells, isolated from the spleen of WT mice or *FASN^+/–^* mice, were stimulated with R848, and the (**T** and **U**) OCR was detected by seahorse, and the (**V**) ATP concentration was detected by ELISA. Murine splenic B cells were pretreated with rmIL-17B for 12 hours and stimulated with R848, and (**W** and **X**) OCR was detected. The data are shown as the mean ± SEM and are representative of 3 independent experiments. *P < 0.05, **P < 0.01, ***P < 0.001, ****P < 0.0001 by 1-way ANOVA with Tukey’s multiple-comparison test (**F** and **G**) or 2-tailed Student’s *t* test (**H**–**L**, **N**–**S**, **U**, **V**, and **X**). NS, *P* > 0.05.

Lipid metabolism plays a key role in regulating the activation and differentiation of B cells (9). To determine whether IL-17B inhibits the activation and differentiation of B cells by regulating lipid metabolism, a lipidomic approach was used to analyze the serum lipid profiles of IMQ-treated WT mice and *IL-17B*–KO mice. The data obtained from the LC-MS analysis were evaluated using principal component analysis (PCA) to visualize the clustering trends of the 2 experimental groups. The samples from the WT mice were scattered in the left region of the PCA plot, whereas the samples from the *IL-17B*–KO mice were scattered in the right region (Figure 4A). Our findings suggested that various lipid metabolites, such as fatty acid (FA) (20:1) and FA(22:5), were significantly upregulated in *IL-17B*–KO mice (Figure 4B and Supplemental Figure 8, A and B). The results of the KEGG pathway analysis demonstrated that these differentially expressed metabolites were enriched in metabolic pathways associated with cholesterol metabolism, fat digestion and absorption, etc. (Figure 4C). FASN, a key enzyme responsible for synthesizing free fatty acids, plays a key role in lipid metabolism. Interestingly, scRNA-seq revealed that the level of FASN in GC B cells was greater in *IL-17B*–KO mice than in WT mice (Figure 4D). Thus, we hypothesized that IL-17B may affect the activation and differentiation of B cells by regulating FASN-mediated lipid metabolism.

To confirm the above hypothesis, we studied the effect of IL-17B on FASN expression and found that IL-17B significantly downregulated FASN expression in a time-dependent and dose-dependent manner in B cells (Figure 4, E–G). We next used mice heterozygous for the FASN gene (*FASN^+/–^*; FASN gene homozygous mouse embryos cannot survive) to determine whether FASN regulates the activation of the TLR and IFN-I pathways in B cells. Splenic naive B cells isolated from WT or *FASN^+/–^* mice were stimulated with LPS, R848, and CpG-1826. As shown in Figure 4, H–J, FASN deficiency strongly inhibited the expression of CD40, CD69, and CD86 induced by LPS, R848, and CpG-1826, as well as the expression of IL-12 (Figure 4K) and TNF-α (Figure 4L). Moreover, FASN deficiency strongly inhibited the R848-induced phosphorylation of the p65, p38, JNK, and Erk proteins (Figure 4M and Supplemental Figure 9, A–D). Additionally, FASN deficiency significantly reversed IFN-α–induced expression of CD40 (Figure 4N), CD69 (Figure 4O), and CD86 (Figure 4P), as well as the IFN-inducible genes MX1 (Figure 4Q), OAS1 (Figure 4R), and IFIT1 (Figure 4S). Notably, the FASN inhibitor TVB-2640 inhibited not only the TLR ligand–induced expression of CD40, CD69, and CD86 (Supplemental Figure 10, A–C) and the phosphorylation of p65 and JNK (Supplemental Figure 10, D–H), but also the IFN-α–induced expression of CD40, CD69, and CD86 (Supplemental Figure 10, I–K). These findings strongly suggested that FASN contributes to the activation of the TLRs and IFN-I signaling pathways in B cells.

The β-oxidation of fatty acids via OXPHOS provides ATP, which is used as an energy source during the activation, differentiation, and proliferation of immune cells, including B cells (11). We next investigated the effect of FASN on mitochondrial OXPHOS and ATP production in B cells. As shown in Figure 4, T–V, compared with those from WT mice, R848-stimulated B cells from *FASN^+/–^* mice exhibited lower levels of mitochondrial OXPHOS and ATP. Consistently, treatment with rmIL-17B considerably decreased the level of mitochondrial OXPHOS in B cells (Figure 4, W and X). We also found that inhibition of OXPHOS by oligomycin significantly inhibited the activation of TLR (Supplemental Figure 11, A–H) and IFN-I signaling pathways (Supplemental Figure 11, I–K) in B cells. These results suggested that IL-17B probably inhibits the activation of TLR and IFN-I signaling pathways through the FASN/OXPHOS axis in B cells.

### FASN contributes to the activation and differentiation of B cells, and the pathogenesis of lupus.

**Figure 5:**
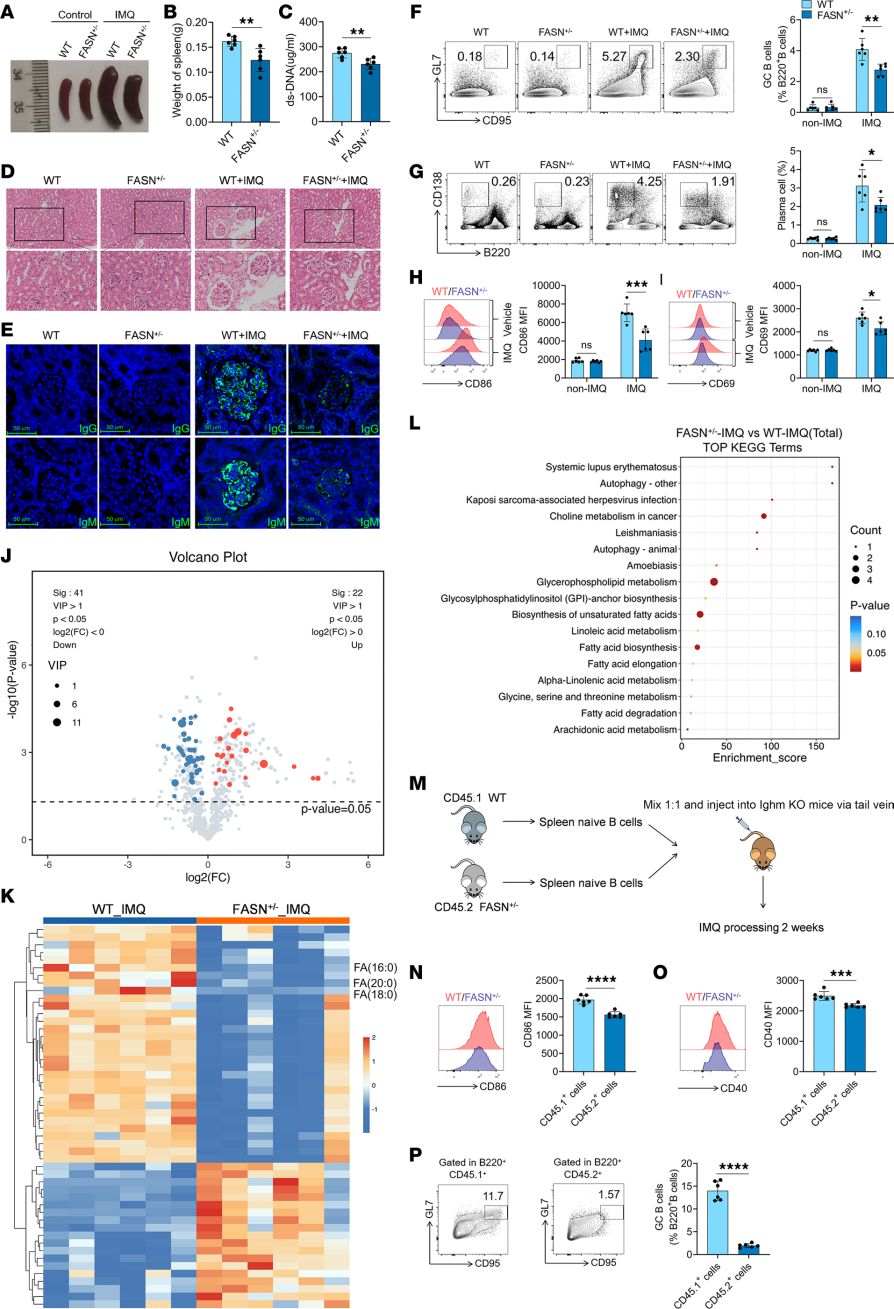
Repression of FASN mitigates IMQ-treated lupus in mice. (**A**) Splenic images, (**B**) spleen weights, (**C**) serum levels of anti-dsDNA antibodies, (**D**) H&E staining of kidney, and (**E**) renal IgG and IgM deposition in WT or *FASN^+/–^* mice treated with IMQ. Scale bars: 100 μm. Representative flow cytometry images and statistical analysis of the percentages of (**F**) spleen GC B cells (B220^+^GL-7^+^CD95^+^), (**G**) plasma cells (CD138^+^B220^–^), and the expression of (**H**) CD86 and (**I**) CD69 on B220^+^ B cells in IMQ-treated WT or *FASN^+/–^* mice. (**J**) Volcano plot, (**K**) heatmap, and (**L**) KEGG pathway analysis based on spleen lipid profiles in IMQ-treated WT mice (*n* = 6) and *FASN^+/–^* mice (*n* = 6). (**M**) Schematic diagram of adoptive transfer. Expression of (**N**) CD86 and (**O**) CD40 on the surface of CD45.1^+^ B cells and CD45.2^+^ B cells in the spleen and the proportion of (**P**) spleen GC B cells (B220^+^GL-7^+^CD95^+^) in CD45.1^+^ B cells and CD45.2^+^ B cells. The data are shown as the mean ± SEM and are representative of 3 independent experiments (*n* = 6 mice/group). *P < 0.05, **P < 0.01, ***P < 0.001, ****P < 0.0001 by 2-tailed Student’s *t* test. NS, *P* > 0.05.

We next investigated the role of FASN in the pathogenesis of lupus. As shown in Figure 5, A–E and Supplemental Figure 12, A–C, compared with WT mice, IMQ-treated *FASN^+/–^* mice exhibited a prominent reduction in the severity of splenomegaly, spleen weights, serum anti-dsDNA antibody levels, kidney injury, and the deposition of IgG and IgM in the glomeruli. More importantly, compared with WT mice, IMQ-treated *FASN^+/–^* mice had a lower proportion of GC B cells and plasma cells and lower levels of CD86 and CD69 expression on B cells in the spleen (Figure 5, F–I) and mLNs (Supplemental Figure 12, D–G). A reduction in the proportions of Tfh and memory CD4^+^ T cells and the expression of CD69 on CD4^+^ T cells in the spleens (Supplemental Figure 12, H–J) and mLNs (Supplemental Figure 12, K and L) of the IMQ-treated *FASN^+/–^* mice was also observed. Because FASN plays a key role in lipid metabolism, a lipidomic approach was used to analyze the lipid profiles of splenocytes from the IMQ-induced *FASN^+/–^* mice and WT mice. As shown in Figure 5, J and K, compared with WT mice, IMQ-treated *FASN^+/–^* mice exhibited significant downregulation of several lipid metabolites, particularly those associated with FASN, such as FA(20:0), FA(18:0), and FA(16:0). The results of the KEGG pathway analysis demonstrated that these differentially expressed metabolites were enriched in metabolic pathways associated with SLE, glycerophospholipid metabolism, biosynthesis of unsaturated fatty acids, and fatty acid biosynthesis (Figure 5L).

To further confirm the role of FASN in the activation and differentiation of B cells, we performed an adoptive transfer experiment. Naive B cells isolated from WT CD45.1 mice or *FASN^+/–^* CD45.2 mice were mixed at a 1:1 ratio and injected into the tail vein of *Ighm*-KO mice. These mice were treated with IMQ for 2 weeks, after which the activation and differentiation of B cells were examined (Figure 5M). Intriguingly, the expression of CD86 and CD40 on CD45.1^+^ B cells was significantly greater than that on CD45.2^+^ B cells in the spleen (Figure 5, N and O) and mLNs (Supplemental Figure 12, M and N). Notably, the proportion of GC B cells among CD45.1^+^ B cells was significantly greater than that among CD45.2^+^ B cells in the spleen (Figure 5P) and mLNs (Supplemental Figure 12O). These results suggest that FASN can directly promote the activation and differentiation of B cells in vivo.

In addition, treatment with TVB-2640 not only ameliorated lupus symptoms in IMQ-induced lupus mice (Supplemental Figure 13, A–F), but also decreased the proportions of GC B cells and plasma cells and the expression of CD86 and CD69 on B cells in the spleen, as well as the proportion of Tfh cells and the expression of CD69 on CD4^+^ T cells in the spleen (Supplemental Figure 13G). Consistently, TVB-2640 had a similar therapeutic effect on MRL/*lpr* mice (Supplemental Figure 14, A–G). These findings indicated that FASN deficiency or targeted inhibition of FASN activity could effectively alleviate the disease in a mouse model of lupus and inhibit the activation and differentiation of B cells.

### SLE B cells showed a lower level of IL-17RB and a greater level of FASN expression.

**Figure 6:**
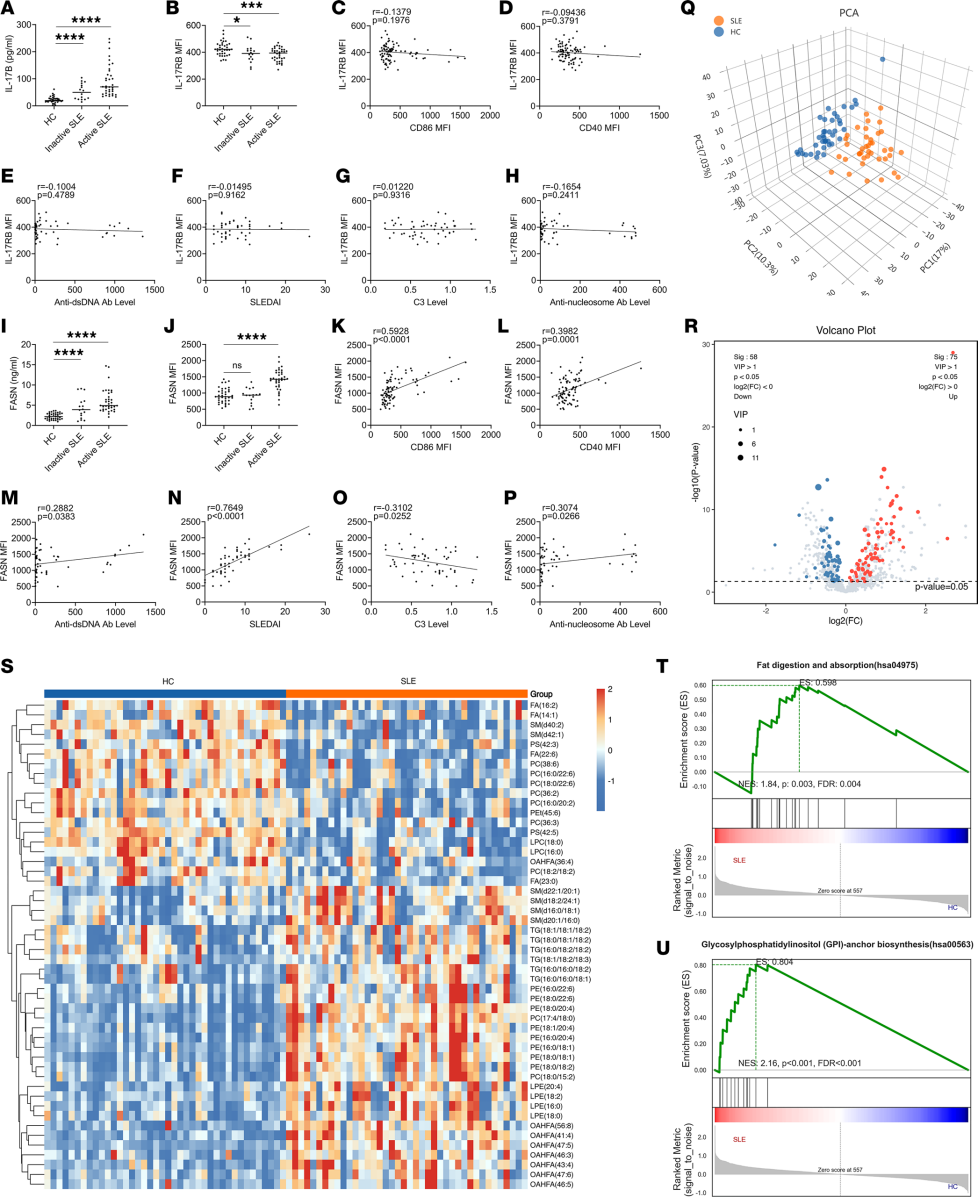
B cells in SLE patients express higher FASN and lower IL-17RB than those in healthy controls. (**A**) Serum IL-17B levels and (**B**) IL-17RB expression on CD19^+^ B cells in healthy controls (HC, *n* = 37), inactive-SLE patients (*n* = 16), and active-SLE patients (*n* = 36). Correlation of IL-17RB with (**C**) CD86 and (**D**) CD40 on CD19^+^ B cells in SLE (*n* = 52) and HC (*n* = 37). Correlation of IL-17RB with levels of (**E**) anti-dsDNA antibody, (**F**) SLEDAI, (**G**) C3 complement, and (**H**) anti-nucleosome antibodies on CD19^+^ B cells with SLE (*n* = 52). (**I**) Serum FASN levels and (**J**) FASN expression on CD19^+^ B cells in HC (*n* = 37), inactive-SLE patients (*n* = 16), and active-SLE patients (*n* = 36). Correlation of FASN with (**K**) CD86 and (**L**) CD40 on CD19^+^ B cells in SLE (*n* = 52) and HC (*n* = 37). Correlation of FASN with levels of (**M**) anti-dsDNA antibodies, (**N**) SLEDAI, (**O**) C3 complement, and (**P**) anti-nucleosome antibodies on CD19^+^ B cells in SLE (*n* = 52). (**Q**) PCA dot plot, (**R**) volcano plot, (**S**) heatmap, and (**T** and **U**) GSEA plots were generated based on the lipid profiles of peripheral blood serum from SLE patients (*n* = 40) and HC (*n* = 40). The data are shown as the mean ± SEM and are representative of 3 independent experiments. *P < 0.05, ***P < 0.001, ****P < 0.0001 by 1-way ANOVA with Tukey’s multiple-comparison test (**A**, **B**, **I**, and **J**). Correlation coefficients were calculated using linear regression analysis. NS, *P* > 0.05.

As IL-17B plays a key role in the pathogenesis of lupus-prone mice, serum and PBMCs from healthy controls, inactive-SLE patients, and active-SLE patients were collected to assess the concentration of IL-17B in serum and the expression of IL-17RB on B cells. As shown in Figure 6, A and B and Supplemental Figure 15, the concentration of serum IL-17B was significantly greater in active-SLE patients than in healthy controls and inactive-SLE patients, while the expression of IL-17RB on B cells was significantly lower in active-SLE patients than in healthy controls and inactive-SLE patients. There was no obvious correlation between the expression of IL-17RB on B cells and the expression of CD86 on B cells (Figure 6C), CD40 expression on B cells (Figure 6D), anti-dsDNA antibody levels (Figure 6E), SLE disease activity index (SLEDAI) score (Figure 6F), C3 complement protein levels (Figure 6G), or anti-nucleosome antibody levels (Figure 6H).

Considering that FASN protein can exist both inside and outside of cells, we first evaluated FASN levels in serum from healthy controls, inactive-SLE patients, and active-SLE patients. As shown in Figure 6I, the level of FASN in serum was significantly greater in active-SLE patients than in healthy individuals and inactive-SLE patients. Similarly, FASN expression was greater in B cells from active-SLE patients than in those from healthy controls and inactive-SLE patients (Figure 6J). Notably, FASN expression in B cells was positively correlated with CD86 and CD40 expression on B cells (Figure 6, K and L), anti-dsDNA antibody levels (Figure 6M), SLEDAI score (Figure 6N), and anti-nucleosome antibody levels (Figure 6P) and was negatively correlated with the C3 complement protein levels (Figure 6O).

As FASN is abnormally highly expressed in SLE patients and lipid metabolism plays a key role in the pathogenesis of SLE, we conducted a comprehensive lipidomic analysis of serum samples obtained from healthy controls and active-SLE patients. As shown in Figure 6Q, the control samples were scattered in the left region of the PCA plot, whereas the SLE samples were scattered in the right region. Our findings suggested that various lipid metabolites, including triglycerides, sphingolipids, lysophosphatidylethanolamines, and phosphatidylethanolamines, were significantly upregulated in patients with SLE (Figure 6, R and S, and Supplemental Figure 16D). KEGG pathway analysis and gene set enrichment analysis (GSEA) demonstrated that these differentially expressed metabolites were enriched in metabolic pathways associated with autophagy, cholesterol metabolism, and fat digestion and absorption, specifically in the context of SLE (Figure 6, T and U, and Supplemental Figure 16, A–C).

In summary, our study showed that IL-17B plays a protective role in the pathogenesis of SLE by inhibiting FASN-mediated activation and differentiation of B cells. Our findings provide insights into what we believe is a novel mechanism underlying the pathogenesis of SLE and provide a strong foundation for the role of IL-17B in the pathogenesis of SLE.

## Discussion

SLE is characterized by a change in self-tolerance, a critical factor in its pathogenesis, where the development of plasma cells plays a key role. In this study, we evaluated whether IL-17B plays a protective role in the pathogenesis of SLE. Interestingly, we found that IL-17B inhibited the activation and differentiation of B cells by downregulating FASN-mediated lipid metabolism and subsequently relieved the onset of SLE.

To date, studies on IL-17B have been limited, and reports on the role of IL-17B in the pathogenesis of immune-related diseases are contradictory. Its function involves the inhibition of intestinal mucosal immunity mediated by IL-17E, and it competes with IL-17E for binding to the receptor IL-17RB (18, 21). However, the regulatory role of IL-17B in SLE is not known. In this study, we found that IL-17B plays a protective role in the pathogenesis of lupus-prone mice by inhibiting the activation and differentiation of B cells. As is known, Tfh cells are necessary for the differentiation of GC B cells. scRNA-seq revealed that the proportion of Tfh cells in the spleens of IMQ-induced *IL-17B*–KO mice was significantly greater than that in the spleens of IMQ-induced WT mice. Thus, determining whether IL-17B modulates the pathogenesis of SLE by regulating the differentiation of Tfh cells and other subsets of T cells is important for elucidating the pathogenesis of SLE.

In the course of conducting the experiment, we did not pay much attention to the effect of IL-17B on the survival rate of MRL/*lpr* mice. The main reason was that the MRL/*lpr* mice used in the experiment were 10–16 weeks old, during which the mortality rate of mice was relatively low, and the effect of IL-17B on the mortality rate could not be compared and analyzed. In order to accurately investigate the effect of IL-17B on the survival rate of MRL/*lpr* mice, it is necessary to redesign the experiment reasonably, such as extending the experimental observation time.

In this study, we used scRNA-seq to reveal the effects of IL-17B on the activation and differentiation of immune cells in the spleens of IMQ-induced lupus mice. Of note, the clustering results of scRNA-seq reveal a substantial abundance of activated and pre-memory B cells in the spleen of mice, while follicular B cells are nearly absent. We suspect that the possible reason may be that the spleens used for scRNA-seq were from IMQ-induced lupus mice. This lupus model mice show a large number of abnormally formed GCs in the spleen, and the abnormal differentiation of B cells may be one of the direct causes of the greater number of activated and pre-memory B cells than follicular B cells.

Lipid metabolism modulates various immune cell populations, thus affecting the progression of autoimmune disorders (22, 23). It has been shown that lipid metabolism is significantly disrupted among individuals diagnosed with SLE (24, 25). In this study, we also detected dyslipidemia in the serum of SLE patients, which highlighted that aberrant lipid metabolism may play an important role in the pathogenesis of SLE by regulating the activation and differentiation of immune cells. Cytoplasmic FASN plays a crucial role in the synthesis of saturated fatty acids, which are necessary for the biosynthesis of lipids and other complex molecules. A study showed that FASN expression was greater in GC B cells than in naive B cells, emphasizing the importance of lipid homeostasis in promoting the proliferation of GC B cells (26). Our study confirmed these findings and provided greater insights into the effect of lipid metabolism on the differentiation of GC B cells. We found that, compared with those from WT mice, B cells from *FASN^+/–^* mice exhibited decreased TLR-induced activation and inflammatory factor production, as well as the capacity to differentiate into GC B cells and plasma cells.

It has been reported that FASN acts as an important proinflammatory factor in macrophages. FASN can enhance the proinflammatory effects of macrophages by promoting the palmitoylation of Akt, which increases the activation of the Akt/MAPK signaling pathway (27). FASN deficiency decreases the retention of plasma membrane cholesterol, thus hindering the transport and activation of Rho GTPase and JNK, which play crucial roles in the transition from M0 to M1 macrophages (28). Considering the critical role of FASN in regulating macrophage polarization, we believe that it is necessary to further study whether FASN is involved in the pathogenesis of SLE through the regulation of macrophage polarization.

In this study, we found that IL-17B could downregulate the expression of FASN in B cells. However, the molecular mechanism of IL-17B regulating FASN expression has not been elucidated. In fact, our research primarily focused on how IL-17B affects the differentiation of GC B cells and the pathogenesis of SLE by regulating the expression of FASN. However, we have not conducted an in-depth exploration of how IL-17B regulates FASN. The regulation of FASN involves a complex network of signal transduction, which controls its gene expression through specific receptors and kinases. In a future study, we will conduct an in-depth investigation to explore whether IL-17B regulates the expression of FASN by influencing related signaling pathways or transcription factors.

Recent studies have revealed a close association between the OXPHOS signaling gene set and specific TLR signaling–related genes (29). Stimulation with R848 was observed to enhance ATP production and OXPHOS in B cells in vitro, yielding findings similar to those previously reported. Fatty acid oxidation is necessary for various cellular reactions, including the catabolism of fatty acids into acetyl-CoA, which enters the tricarboxylic acid cycle to facilitate ATP production (30). In this study, we found that rmIL-17B treatment and FASN deficiency led to decreased OXPHOS capacity and ATP production in R848-stimulated B cells in vitro. We also found that oligomycin, a potent inhibitor of OXPHOS, significantly inhibited the activation of the TLR signaling pathway in B cells in vitro. Overall, our findings highlighted that lipid metabolism and mitochondrial energy production play key roles in facilitating TLR-mediated activation and differentiation of B cells.

Although serum IL-17B levels were found to increase in SLE patients, we also recorded a concurrent decrease in IL-17RB on B cells within this patient population. However, the expression of IL-17RB on B cells from SLE patients was not correlated with CD86 and CD40 levels, anti-dsDNA antibodies, C3 complement, SLEDAI scores, and the level of anti-nucleosome antibodies. We speculate that the high expression of IL-17B in SLE patients might be a feedback protection mechanism in the body. Thus, the mechanism underlying the high level of IL-17B expression in SLE patients needs to be further investigated. We also speculate that a certain balance might exist between the high expression of IL-17B in serum and the low expression of IL-17RB in B cells.

In summary, we report what we believe is a previously unknown function of IL-17B. Our study revealed that IL-17B plays a crucial protective role in the pathogenesis of SLE. IL-17B inhibits the activation and differentiation of B cells by regulating FASN-mediated lipid metabolism, thus alleviating the pathogenesis of SLE. Overall, our finds provide a solid foundation for comprehending the involvement of IL-17B in SLE.

## Methods

### Sex as a biological variable.

All experimental mice were female, as female mice demonstrate more pronounced lesions in the induced lupus model. The patients with SLE enrolled in this study were all female, because SLE tends to occur in young women.

### Mice.

Female C57BL/6 mice (6–8 weeks old) were purchased from Pengyue Experimental Animal Breeding Co., Ltd. Female MRL-fas*^lpr^* (MRL/*lpr*) mice (8 weeks old) were purchased from Aniphe Biolaboratory, Inc. *IL-17B*^–/–^ mice were purchased from GemPharmatech Co., Ltd., and *FASN^+/–^* mice were purchased from Cyagen Biosciences Inc. All mice were bred and maintained at Jining Medical University under specific pathogen–free (SPF) conditions, following a 12-hour light/12-hour dark cycle, and fed a standard chow diet. All animal experiments complied with the ARRIVE guidelines.

### Information on patients and healthy donors.

All SLE patients and healthy donors provided written informed consent before the study. Peripheral blood samples were collected from SLE patients and healthy donors. All SLE patients were diagnosed following the criteria set by the American College of Rheumatology revised criteria (1997). Whole blood samples from healthy donors and SLE patients were collected. Although the patients were on various disease-modifying agents, we excluded only those patients who were administered high-dose immunocytotoxic therapeutic agents or steroids from the study. Patients who had an overlapping syndrome were also excluded from the study. Disease activity was evaluated using the SLEDAI, and a cutoff of 5 or greater was used to define active disease.

### Animal experiments.

Before starting animal experiments, we worked out a detailed protocol. To construct an IMQ-induced lupus-prone mouse model, female C57BL/6, *IL-17B^–/–^* or *FASN^+/–^* mice (8 weeks old, *n* = 6) were treated with 1.25 mg of 5% IMQ cream (Sichuan MED-SHINE Pharmaceutical Co., Ltd.), which was applied to the ear 3 times a week. After 10 weeks of treatment, all mice were sacrificed by inhalation of carbon dioxide, and their spleens, mLNs, kidneys, and blood were collected for further analysis.

To explore the effect of rmIL-17B on the pathogenesis of MRL/*lpr* mice, female MRL/*lpr* mice (10 weeks old) were randomly divided into 2 groups: one group (*n* = 6) was injected with rmIL-17B, the other group (*n* = 6) was injected with vehicle. rmIL-17B (0.5 μg/g; MCE) or vehicle was injected into mice via the tail vein (twice per week). After 6 weeks of treatment, all mice were sacrificed by inhalation of carbon dioxide for further analysis. To explore the effect of rmIL-17B on the pathogenesis of IMQ-induced lupus-prone mice, female C57BL/6 mice (8 weeks old) were randomly divided into 4 groups: mice from group A (*n* = 6) were injected with vehicle, mice from group B (*n* = 6) were injected with rmIL-17B, mice from group C (*n* = 6) were treated with IMQ and injected with vehicle, and mice from group D (*n* = 6) were treated with IMQ and injected with rmIL-17B. rmIL-17B (0.5 μg/g) or vehicle was injected into mice via the tail vein (twice per week). After 10 weeks of treatment, all mice were sacrificed by inhalation of carbon dioxide for further analysis. To explore the effect of TVB-2640 on the pathogenesis of IMQ-induced lupus-prone mice, female C57BL/6 mice (8 weeks old) were randomly divided into 4 groups: mice from group A (*n* = 6) were injected with vehicle, mice from group B (*n* = 6) were injected with TVB-2640, mice from group C (*n* = 6) were treated with IMQ and injected with vehicle, and mice from group D (*n* = 6) were treated with IMQ and injected with TVB-2640. TVB-2640 (20 μg/g; Selleck) or vehicle was injected into mice intraperitoneally (twice per week). After 10 weeks of treatment, all mice were sacrificed by inhalation of carbon dioxide for further analysis. To explore the effect of TVB-2640 on the pathogenesis of MRL/*lpr* mice, female MRL/*lpr* mice (10 weeks old) were randomly divided into 2 groups: one group (*n* = 6) was injected with TVB-2640, the other group (*n* = 6) was injected with vehicle. TVB-2640 (20 μg/g) or vehicle was injected into mice intraperitoneally (twice per week). After 6 weeks of treatment, all mice were sacrificed by inhalation of carbon dioxide for further analysis. To explore the effects of rmIL-17B on SRBC-immunized mice, female C57BL/6 mice (8 weeks old) were randomly divided into 3 groups. Group A mice (*n* = 6) were not treated, group B mice (*n* = 6) were injected with SRBCs and vehicle, and group C mice (*n* = 6) were injected with SRBCs and rmIL-17B. SRBCs (4 × 10^8^ cells/mouse) were injected intraperitoneally on the first day. In addition, rmIL-17B (0.5 μg/g) was injected into the mice via the tail vein (3 times per week). After 2 weeks of treatment, all mice were sacrificed by inhalation of carbon dioxide for further analysis.

### Isolation of human PBMCs.

After collecting blood samples from SLE patients and healthy participants in heparinized tubes, PBMCs were isolated using Ficoll-Paque PLUS (GE Healthcare).

### scRNA-seq.

Splenic tissues from IMQ-treated *IL-17B*–KO and WT mice were used to prepare single-cell suspensions, which were subsequently sent to OE Biotech for scRNA-seq. After dissociation into single-cell suspensions, 10× Genomics single-cell transcriptome sequencing was performed. The preliminary CellRanger quality control results were analyzed using the Seurat software package (https://satijalab.org/seurat/articles/install_v5.html) for further quality control and treatment. After quality control, we included 2 groups of 52,214 cells for further analysis.

### Lipidomics.

Splenocytes from 6 mice per group were quickly frozen in liquid nitrogen and sent to Shanghai Luming Biological Technology Co., Ltd. for LC-MS/MS–based lipid mediator lipidomics. An ACQUITY UPLC I-Class plus (Waters Corporation) fitted with a Q Exactive mass spectrometer equipped with a heated electrospray ionization (ESI) source (Thermo Fisher Scientific) was used for metabolic profiling in both ESI-positive and ESI-negative ion modes. The original Q Exactive LC-MS/MS data in raw format were processed by the software LipidSearch for MSn (Thermo Fisher Scientific), and the exact mass-charge ratio (*m*/*z*) of the parent ions was determined. Differentially abundant metabolites with variable importance in projection values greater than 1.0 and *P* values less than 0.05 were selected.

### Antibodies.

The antibodies used for immunoblotting included anti-p38 (catalog 8690), anti–p-p38 (catalog 4511), anti-Erk (catalog 4695), anti–p-Erk (catalog 4370), anti-JNK (catalog 9252), anti–p-JNK (catalog 4668), anti-p65 (catalog 8242), and anti–p-p65 (catalog 3033). These antibodies were purchased from Cell Signaling Technology and used at a 1:1000 dilution. Anti-FASN (catalog ab128870, diluted 1:1000) was purchased from Abcam. Anti–β-actin (catalog AA128, diluted 1:1000), HRP-labeled goat anti-rabbit antibodies (catalog A0208, diluted 1:3000), and HRP-labeled goat anti-mouse antibodies (catalog A0216, diluted 1:3000) were purchased from Beyotime Institute of Biotechnology. The flow cytometry experiments were conducted using antibodies purchased from BioLegend. These antibodies included FITC-labeled anti–mouse B220 (catalog 103206) and CD4 (catalog 100406); PE-labeled anti–mouse CD40 (catalog 124610), GL7 (catalog 144607), PD-1 (catalog 135206), and CD44 (catalog 103007); APC-labeled anti–mouse CD86 (catalog 105012), CD95 (catalog 152604), CXCR5 (catalog 145506), and CD138 (catalog 142506); BV421-labeled anti–mouse CD69 (catalog 104528) and CD62L (catalog 104436); PE-labeled anti–human CD40 (catalog 334308); APC-labeled anti–human CD86 (catalog 374208); and APC/Cy7-labeled anti–human CD19 (catalog 302218). APC-labeled anti–human IL-17RB (catalog FAB1207A) was purchased from R&D Systems, and APC-labeled anti–human FASN (catalog ab223965) was purchased from Abcam. An isotype control was used for each antibody. The antibodies used for confocal immunofluorescence microscopy were Alexa Fluor 488–conjugated goat anti–mouse IgG (catalog A11017, Invitrogen) and Alexa Fluor 488–conjugated goat anti–mouse IgM (catalog A21042, Invitrogen).

### Isolation of murine splenic B cells.

Naive B cells were isolated from the spleens of mice via a negative selection approach using a mouse B cell isolation kit (BD); the isolated cells were greater than 95% pure. The sorted B cells were subsequently cultured in RPMI 1640 medium supplemented with 10% fetal bovine serum (both Gibco) for further analysis.

### Adoptive transfer study.

Murine naive B cell suspensions from WT mice (CD45.1) or *FASN^+/–^* mice (CD45.2) were mixed at a 1:1 ratio and intravenously injected into *Ighm*-KO mice (*n* = 6) (GemPharmatech Co., Ltd). Subsequently, IMQ was topically applied to the right ear of each mouse 3 times a week. After 2 weeks, the mice were euthanized and experiments were conducted.

### H&E staining.

Mouse kidney tissues were fixed with paraformaldehyde, dehydrated in ethanol, and embedded in paraffin. Tissue sections (5 μm thick) were stained with a 0.1% hematoxylin solution for 10 minutes, followed by treatment with a 0.5% eosin solution for 1 minute at 22°C ± 2°C. Histopathological changes were observed under an optical microscope (Nikon Corporation) within randomly selected fields of view. Blind assessment of semiquantitative pathological changes was conducted, focusing primarily on the 15 cortical glomeruli of each mouse. In summary, glomerulonephritis activity is graded from 0 to 3 based on the degree of glomerular cell proliferation and leukocyte extravasation: 0, no proliferation; 1, <25%; 2, 25%–50%; and 3, >50% of glomeruli (31).

### Immunofluorescent staining.

Kidney tissue sections were first dewaxed and rehydrated using a xylene-ethanol mixture, followed by incubation with 1% BSA to block nonspecific binding. Next, the tissue sections were incubated overnight at 4°C with Alexa Fluor 488–labeled goat anti–mouse IgG or IgM. The following day, the sections were washed with PBS containing 0.1% Tween 20 and stained with DAPI. After staining, the tissue sections were placed under coverslips using Antifade Mounting Medium (Beyotime). Finally, the coverslips were sealed with an fluorescence-quenching agent, and the sections were viewed under a fluorescence microscope (Olympus). Blind assessment of semiquantitative pathological changes was conducted, focusing primarily on the 15 cortical glomeruli of each mouse. In summary, the degree of IgG or IgM deposition in the glomerulus is assessed by evaluating the intensity of IgG or IgM staining, using the following specific grading criteria: 0, no staining; 1 (weak), <25%; 2 (moderate), 25%–50%; or 3 (strong), >50% of glomeruli (31).

### Quantitative real-time PCR.

TRIzol reagent (Invitrogen) was used to extract total RNA from cells. A RevertAid First Strand cDNA Synthesis Kit (Thermo Fisher Scientific) was used to synthesize cDNA from the isolated total RNA. SYBR Green PCR Master Mix (Vazyme Biotech) was used to amplify the obtained cDNA. The level of mRNA expression was normalized to the expression of *Gapdh* mRNA. The 2^−ΔΔCt^ method was used to quantify the fold change in gene expression.

### Flow cytometry.

The cells were collected and transferred to tubes. After washing twice with PBS, an appropriate volume of flow cytometry antibody was added. Next, the cells were incubated at 4°C for 30 minutes, washed twice with PBS, and analyzed using a BD FACSVerse instrument. All FACS data were processed using FlowJo software. To ensure that the results were accurate, an isotype control was included for each antibody used in the analysis.

### ELISA.

The level of IL-17B in the serum of SLE patients and healthy individuals was determined using a human IL-17B ELISA Kit (Shanghai JONLN Reagent Co., Ltd.) following standard procedures. The level of anti-dsDNA IgG was analyzed using a mouse anti-dsDNA IgG Kit (Bethyl Laboratories) following standard procedures. A microplate reader (BioTek) was used to measure the absorbance at 450 nm. All the samples were analyzed in duplicate.

### Immunoblotting analysis.

Protein lysates (60 μg) were resolved by electrophoresis in 7.5%–12% SDS-PAGE gels. After separation, the proteins were transferred onto polyvinylidene difluoride (PVDF) membranes using a transfer solution at a voltage of 100 V for 1 hour. After the proteins were transferred, the membranes were blocked with a solution containing 3% BSA and incubated at room temperature for 2 hours. Next, primary antibodies (diluted 1:1000) were added and incubated overnight at 4°C. The following day, the membranes were washed thoroughly and treated with HRP-conjugated secondary antibodies. Finally, chemiluminescence detection was performed using an ECL kit (Thermo Fisher Scientific) to visualize the protein bands. ImageJ software (NIH) was employed for the quantification of band intensities, followed by normalization of all protein expression levels.

### Oxygen consumption rate.

A Seahorse XFe24 extracellular flux analyzer (Agilent) was utilized to measure the rate of oxidative consumption (OCR). Briefly, 24-well Seahorse plates (Agilent) coated with poly(L-lysine) (Sigma-Aldrich) were prepared with 1 × 10^5^ B cells per well. The plates were subsequently placed in a temperature-controlled environment at 37°C for 1 hour, devoid of any CO_2_. The mitochondrial respiratory inhibitors were applied to the plates in a sequential manner: oligomycin was administered at a concentration of 1 μM, followed by the protonophore carbonyl cyanide 4-(trifluoromethoxy)phenylhydrazone (FCCP) at a concentration of 10 μM, and finally a combination of rotenone and antimycin A at a concentration of 1 μM.

### Statistics.

All the statistical analyses were performed using Prism software (GraphPad). Statistical analyses were conducted using a 2-tailed, unpaired Student’s *t* test, 1-way ANOVA with Tukey’s multiple-comparison test, or 2-way ANOVA with Bonferroni’s multiple-comparison test to assess any variations among the groups. Pearson’s correlation analysis was used to determine the relationship of paired data. All differences were considered to be statistically significant at a *P* value of less than 0.05. Correlation coefficients were calculated using linear regression analysis.

### Study approval.

All experiments were conducted according to institutional guidelines for animal care and the guide for the Animal Care Committee of Jining Medical University (JNMC-2023-DW-136). Before collecting samples, all enrolled patients provided their signature on an informed consent form. The study protocol was approved by the Research Ethics Committee of Jining Medical University (JNMC-2023-YX-018).

### Data availability.

The scRNA-seq data from this study have been deposited in the China National GeneBank (CNGB) Nucleotide Sequence Archive (CNSA) (https://db.cngb.org/cnsa/) with accession number CNP0005844. All the values of the data points in the graph are available in the supplemental Support Data Values file.

## Author contributions

All authors contributed to the study conception and design. YY, HX, and GD engaged in study design and coordination and material support for obtained funding, and supervised the study. YX, YY, YH, YG, LW, LZ, QM, ZN, and LY performed most of the experiments and statistical analyses and wrote the manuscript. HL, JL, and JW performed parts of the experiments. All authors reviewed and approved the final manuscript.

## Supplementary Material

Supplemental data

Unedited blot and gel images

Supporting data values

## Version 1 08/08/2024

In-Press Preview

## Version 2 09/24/2024

Electronic publication
