# Supplementary material for: IL-17B alleviates the pathogenesis of systemic lupus erythematosus by inhibiting FASN-mediated differentiation of B cells
Source: JCI Insight. 2024 Aug 8;9(18):e181906. doi: 10.1172/jci.insight.181906 (PMC11457847; doi:10.1172/jci.insight.181906)
Supplement: Supplemental data [file jciinsight-9-181906-s091.pdf]

Supplemental Data

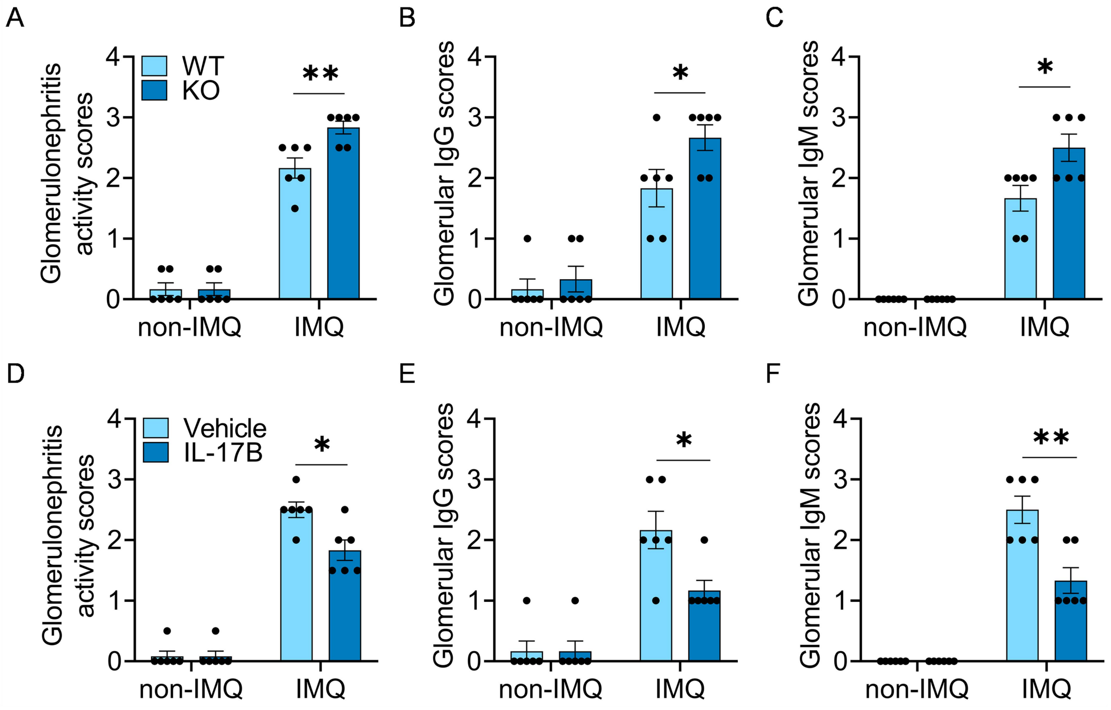

Supplemental Figure 1. IL-17B alleviate the pathological damage to the kidneys induced by IMQ in mice. (**A**) Glomerulonephritis activity scores, (**B**) glomerular IgG scores and (**C**) glomerular IgM scores in wild-type or *IL-17B* KO mice treated with IMQ. (**D**) Glomerulonephritis activity scores, (**E**) glomerular IgG scores and (**F**) glomerular IgM scores in IMQ-induced wild-type mice treated with vehicle or rmIL-17B. The data are shown as the means ± SEM and are representative of three independent experiments (n=6 mice/group). **p* < 0.05, ***p* < 0.01, and as determined by two-way ANOVA.

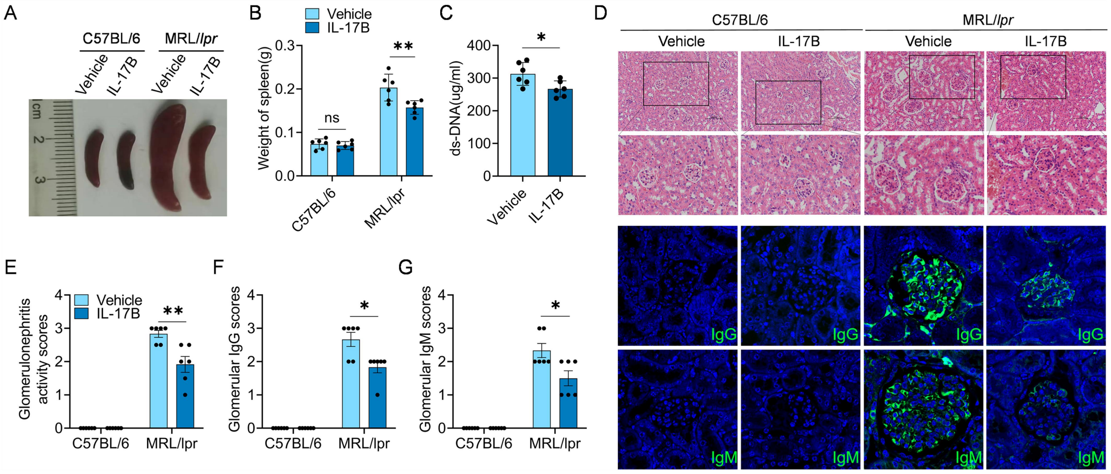

Supplemental Figure 2. IL-17B inhibits disease progression in MRL/*lpr* mice. (**A**) Splenic images, (**B**) spleen weights, (**C**) serum levels of anti-dsDNA antibodies, (**D-G**) H&E staining of kidney, and renal IgG and IgM deposition in MRL/*lpr* mice treated with vehicle or rmIL-17B. Scale bars represent 100 μm. The data are shown as the means ± SEM and are representative of three independent experiments (n=6 mice/group). *$p < 0.05$, **$p < 0.01$, ns denotes $p > 0.05$, and as determined by two-tailed Student's *t*-test or two-way ANOVA.

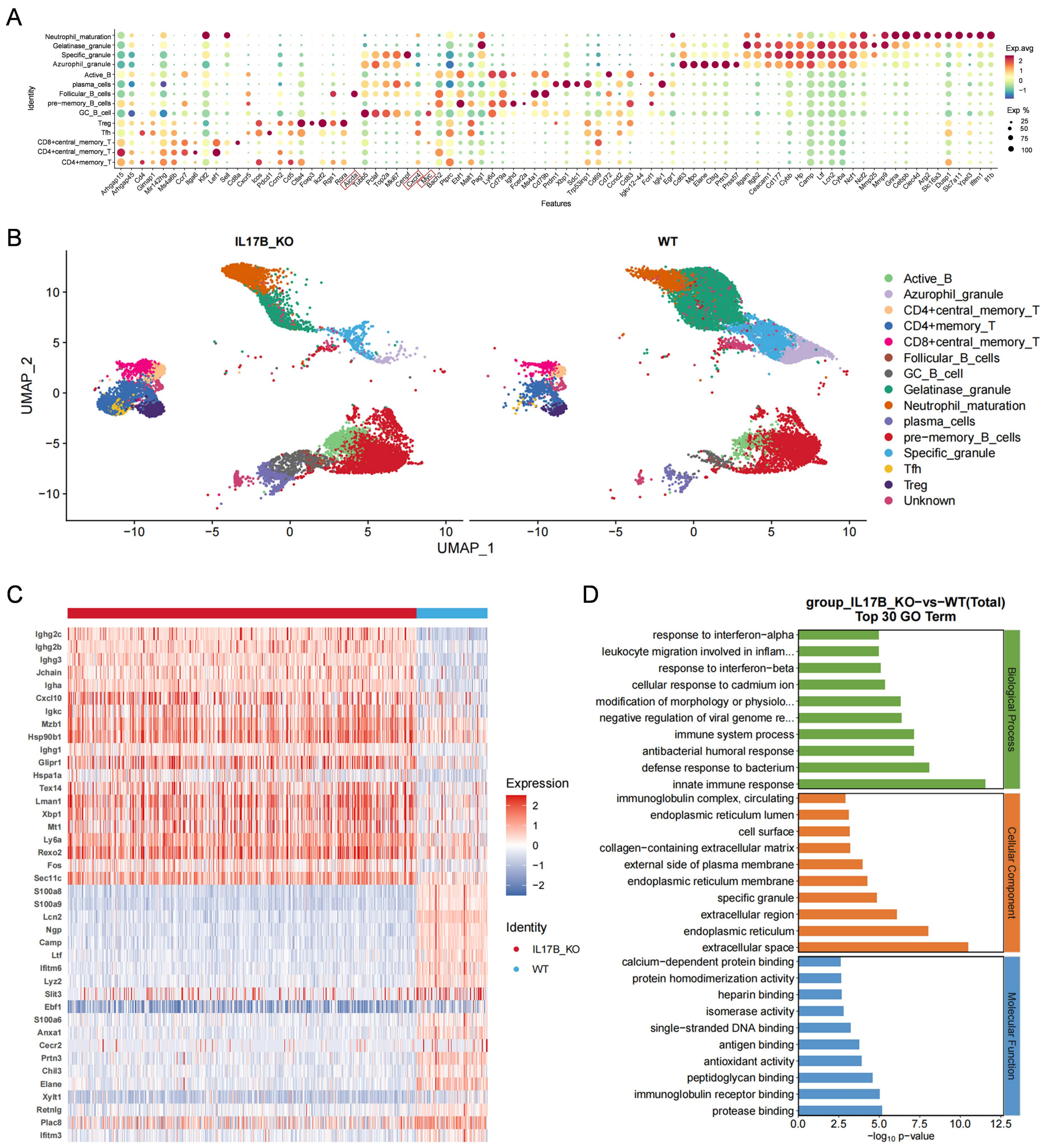

Supplemental Figure 3. Single-cell RNA sequencing analysis of IMQ-treated wild-type mice and *IL-17B* KO mice. (**A**) Dot plots depict the mean expression level of a known marker within a specific cell cluster, with dot size reflecting the proportion of cells in each cluster expressing the gene. (**B**) UAMP plots of spleen cells from IMQ-treated wild-type mice (n=2) and *IL-17B* KO mice (n=2). (**C**) Heatmap and (**D**) GO analysis of spleen GC B cells from IMQ-treated wild-type mice and *IL-17B* KO mice.

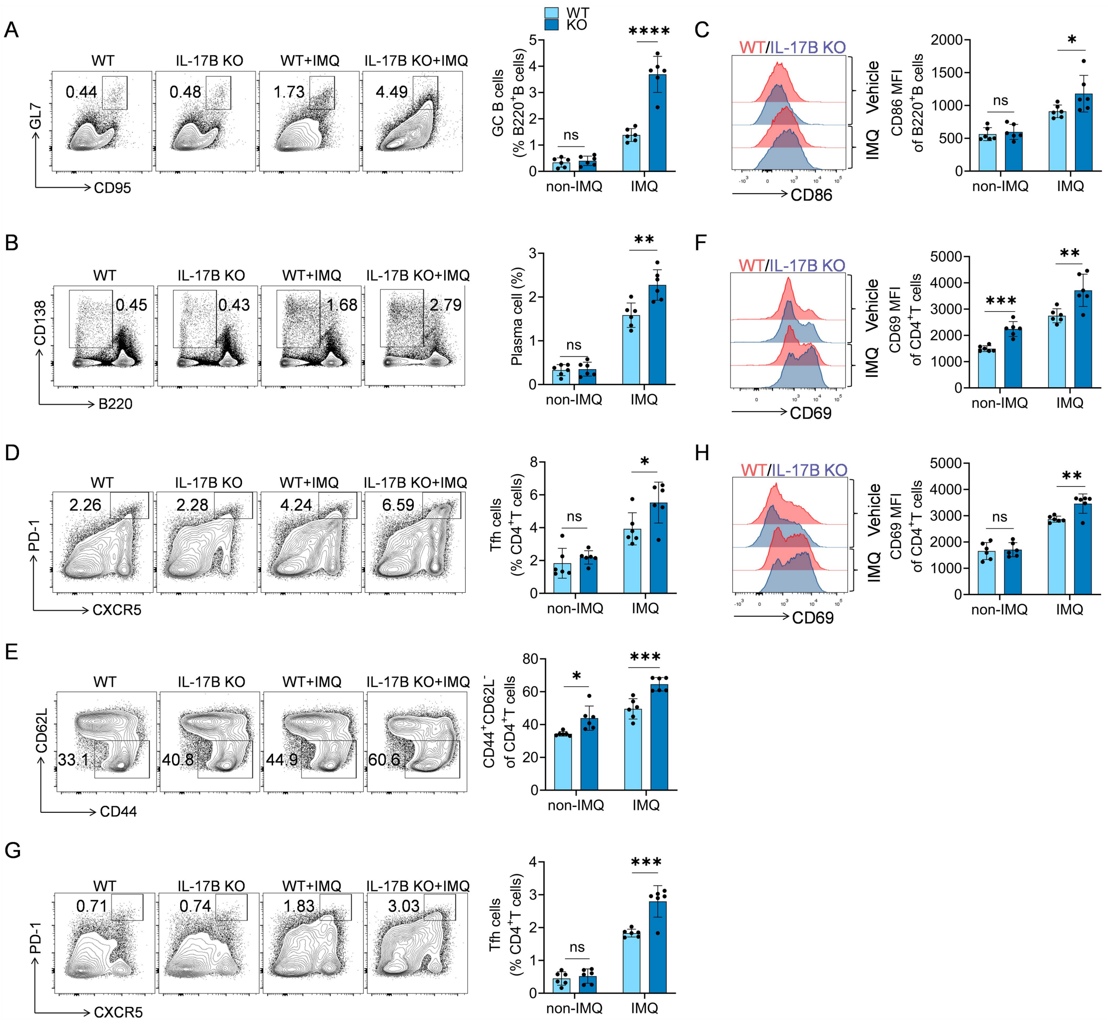

Supplemental Figure 4. Deletion of IL-17B promotes the activation and differentiation of B cells and T cells from mLNs of IMQ-induced lupus-prone mice. Representative flow cytometry images and statistical analysis of the percentage of (**A**) GC B cells, (**B**) plasma cells, and the expression of (**C**) CD86 on B220^{+} B cells in mLNs of IMQ-treated wild-type or *IL-17B* KO mice. Representative flow cytometry images and statistical analysis of the percentage of **(D)** Tfh cells, **(E)** memory CD4^{+} T cells and the expression of **(F)** CD69 on CD4^{+} T cells in spleen and the percentage of **(G)** Tfh cells and the expression of **(H)** CD69 on CD4^{+} T cells in the mLNs of IMQ-treated wild-type or *IL-17B* KO mice. The data are shown as the means ± SEM and are representative of three independent experiments (n=6 mice/group). *$p < 0.05$, **$p < 0.01$, ***$p < 0.001$, ****$p < 0.0001$, ns denotes $p > 0.05$, and as determined by two-tailed Student's *t*-test.

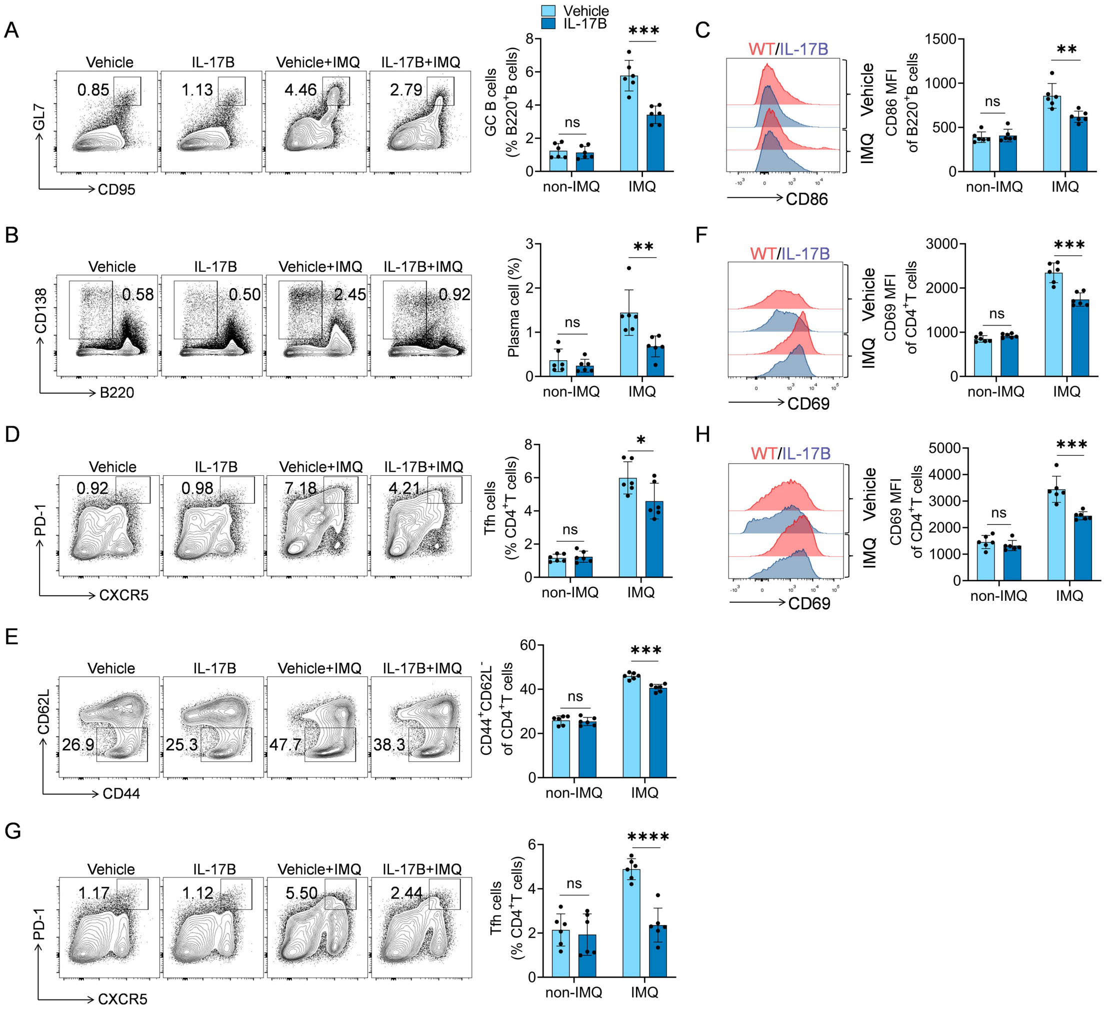

Supplemental Figure 5. rmIL-17B inhibits the activation and differentiation of B cells and T cells. Representative flow cytometry images and statistical analysis of the percentage of (**A**) GC B cells, (**B**) plasma cells, and the expression of (**C**) CD86 on B220$^+$ B cells in mLNs of IMQ-induced wild-type mice treated with vehicle or rmIL-17B. Representative flow cytometry images and statistical analysis of the percentage of **(D)** Tfh cells, **(E)** memory CD4$^+$ T cells and the expression of **(F)** CD69 on CD4$^+$ T cells in spleen and the percentage of **(G)** Tfh cells and the expression of **(H)** CD69 on CD4$^+$ T cells in the mLNs of IMQ-induced wild-type mice treated with vehicle or rmIL-17B. The data are shown as the means ± SEM and are representative of three independent experiments (n=6 mice/group). *p < 0.05, **p < 0.01, ***p < 0.001, ****p < 0.0001, ns denotes p > 0.05, and as determined by two-tailed Student's t-test.

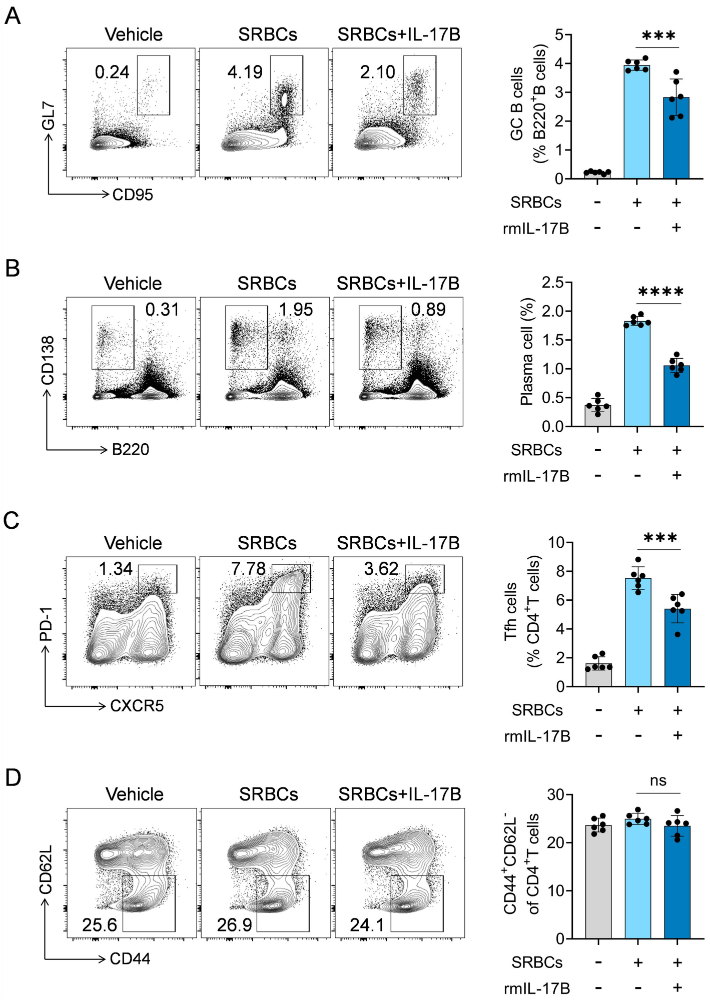

Supplemental Figure 6. IL-17B inhibits the germinal center response induced by exogenous antigens. Representative flow cytometry images and statistical analysis of the percentages of (**A**) spleen GC B cell, (**B**) plasma cells, (**C**) Tfh cells, **(D)** memory $CD4^+$ T cells in SRBCs-induced wild-type mice treated with vehicle or rmIL-17B. The data are shown as the means ± SEM and are representative of three independent experiments (n=6 mice/group). ***p < 0.001, ****p < 0.0001, ns denotes p > 0.05, and as determined by one-way ANOVA.

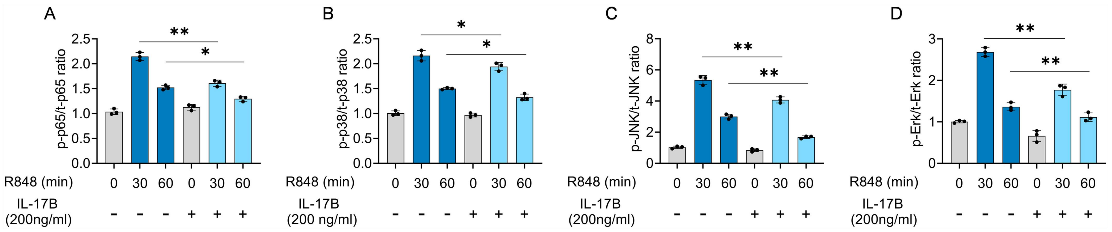

Supplemental Figure 7. IL-17B reduces the phosphorylation levels of p65, p38, JNK, and Erk induced by R848. Normalized quantification of phosphorylation levels of (**A**) p65, (**B**) p38, (**C**) JNK, and (**D**) Erk protein in Figure 3F. The data are shown as the means ± SEM and are representative of three independent experiments. *$p < 0.05$, **$p < 0.01$, and as determined by two-way ANOVA.

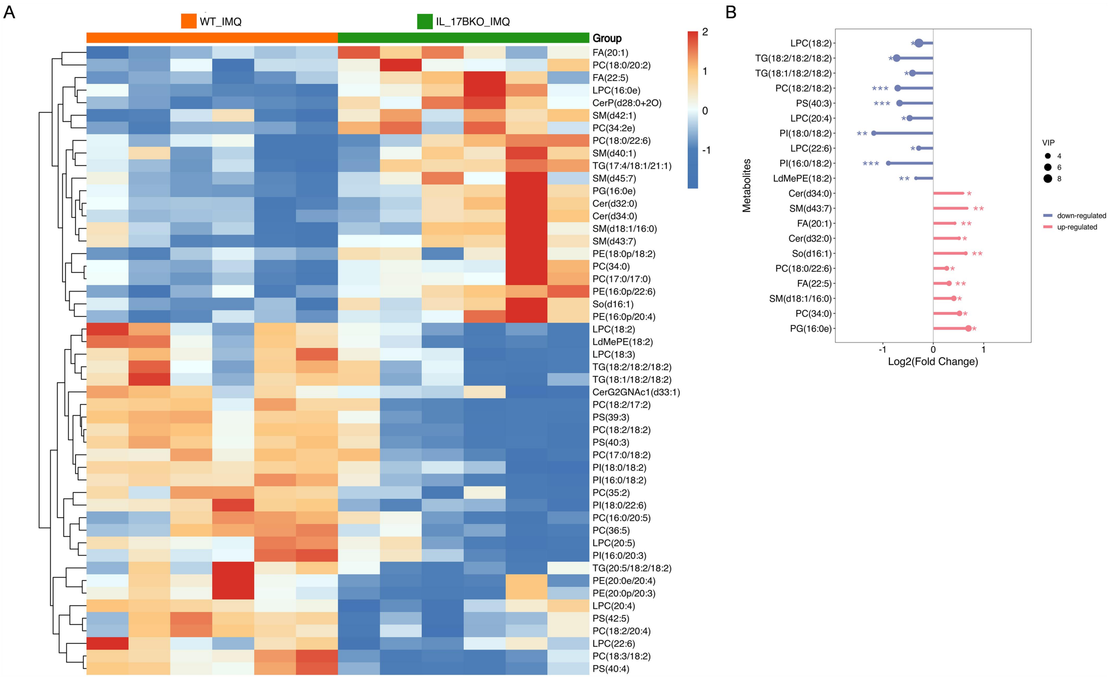

Supplemental Figure 8. Liposome profiling of IMQ-treated wide-type and *IL-17B* KO mice. (**A**) Heatmaps and (**B**) lollipops of metabolites from serum lipid profiles of IMQ-treated wild-type (n=6) and *IL-17B* KO mice (n=6).

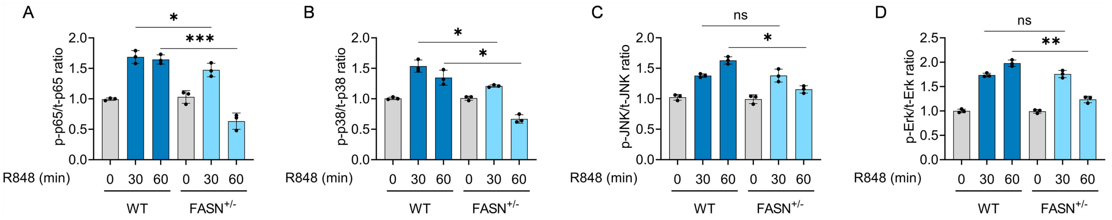

Supplemental Figure 9. Absence of FASN reduces the phosphorylation levels of p65, p38, JNK, and Erk induced by R848. Normalized quantification of phosphorylation levels of (**A**) p65, (**B**) p38, (**C**) JNK, and (**D**) Erk protein in Figure 4M. The data are shown as the means ± SEM and are representative of three independent experiments. *$p < 0.05$, **$p < 0.01$, ***$p < 0.001$, ns denotes $p > 0.05$, and as determined by two-way ANOVA.

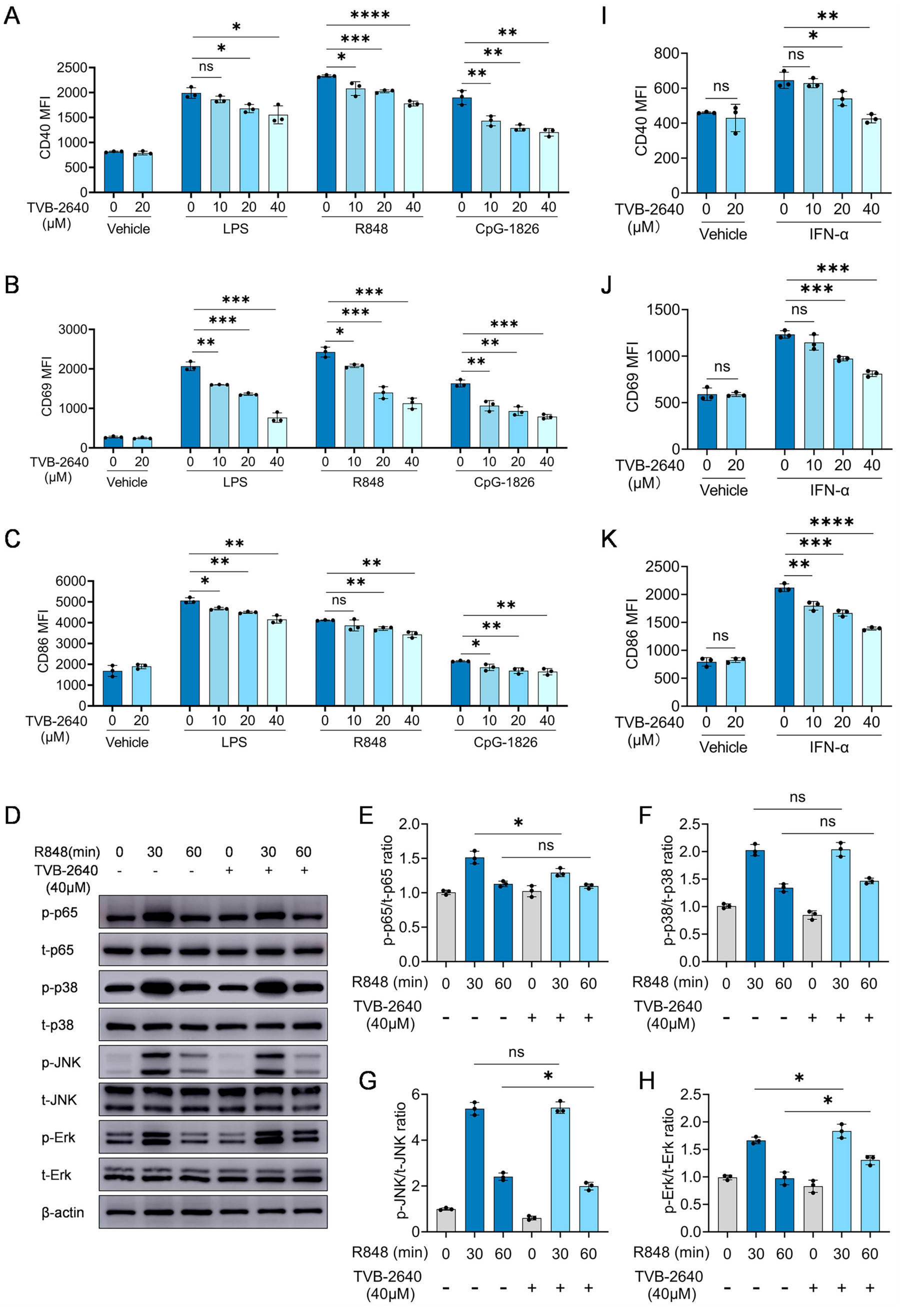

Supplemental Figure 10. TVB-2640 suppresses TLR and IFN-I-induced B cell activation in vitro. (A-H) Murine splenic naive B cells, pretreated with TVB-2640 for 3 hours, were stimulated with LPS (100 ng/ml), R848 (1 μg/ml) and CpG-1826 (1 μM). (**A**) CD40, (**B**) CD69 and (**C**) CD86 expression at 24 hours. (**D-H**) Western blot analysis was performed to assess the phosphorylation levels of p65, p38, JNK, and Erk at 30 or 60 minutes after R848 stimulation. **(I-K)** Murine splenic naive B cells, pretreated with TVB-2640 for 3 hours, were stimulated with IFN-α (1000 U/ml). (**I**) CD40, (**J**) CD69 and (**K**) CD86 expression at 24 hours. The data are shown as the means ± SEM and are representative of three independent experiments. *$p < 0.05$, **$p < 0.01$, ***$p < 0.001$, ****$p < 0.0001$, ns denotes $p > 0.05$, and as determined by one-way or two-way ANOVA.

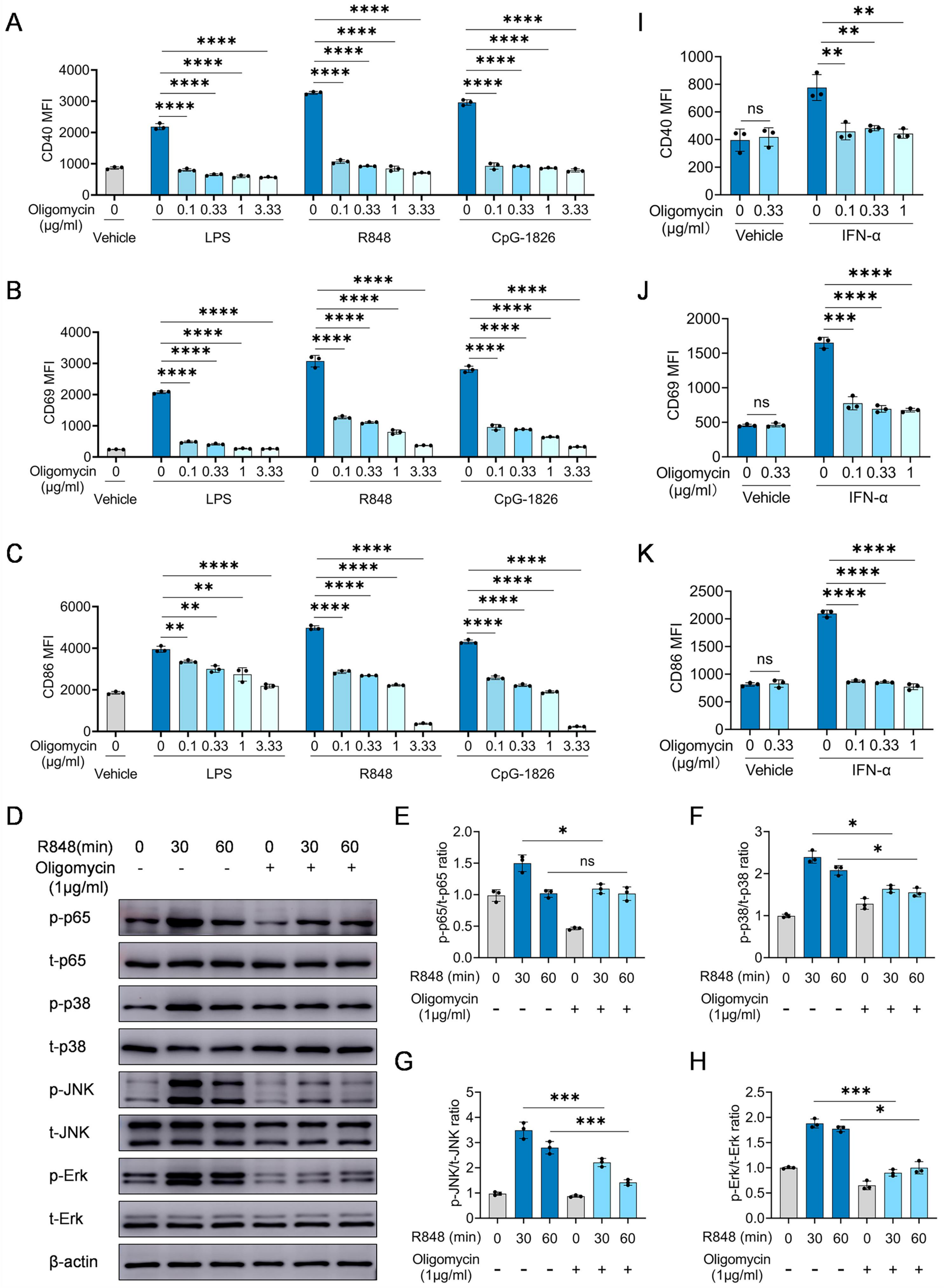

Supplemental Figure 11. Oligomycin suppresses TLR and IFN-I-induced B cell activation in vitro. (A-H) Murine splenic naive B cells, pretreated with Oligomycin for 2 hours, were stimulated with LPS (100 ng/ml), R848 (1 μg/ml) and CpG-1826 (1 μM). (**A**) CD40, (**B**) CD69 and (**C**) CD86 expression at 24 hours. (**D-H**) Western blot analysis was performed to assess the phosphorylation levels of p65, p38, JNK, and Erk at 30 or 60 minutes after R848 stimulation. **(I-K)** Murine splenic naive B cells, pretreated with Oligomycin for 2 hours, were stimulated with IFN-α (1000 U/ml). (**I**) CD40, (**J**) CD69 and (**K**) CD86 expression at 24 hours. The data are shown as the means ± SEM and are representative of three independent experiments. *p < 0.05, **p < 0.01, ***p < 0.001, ****p < 0.0001, ns denotes p > 0.05, and as determined by one-way or two-way ANOVA.

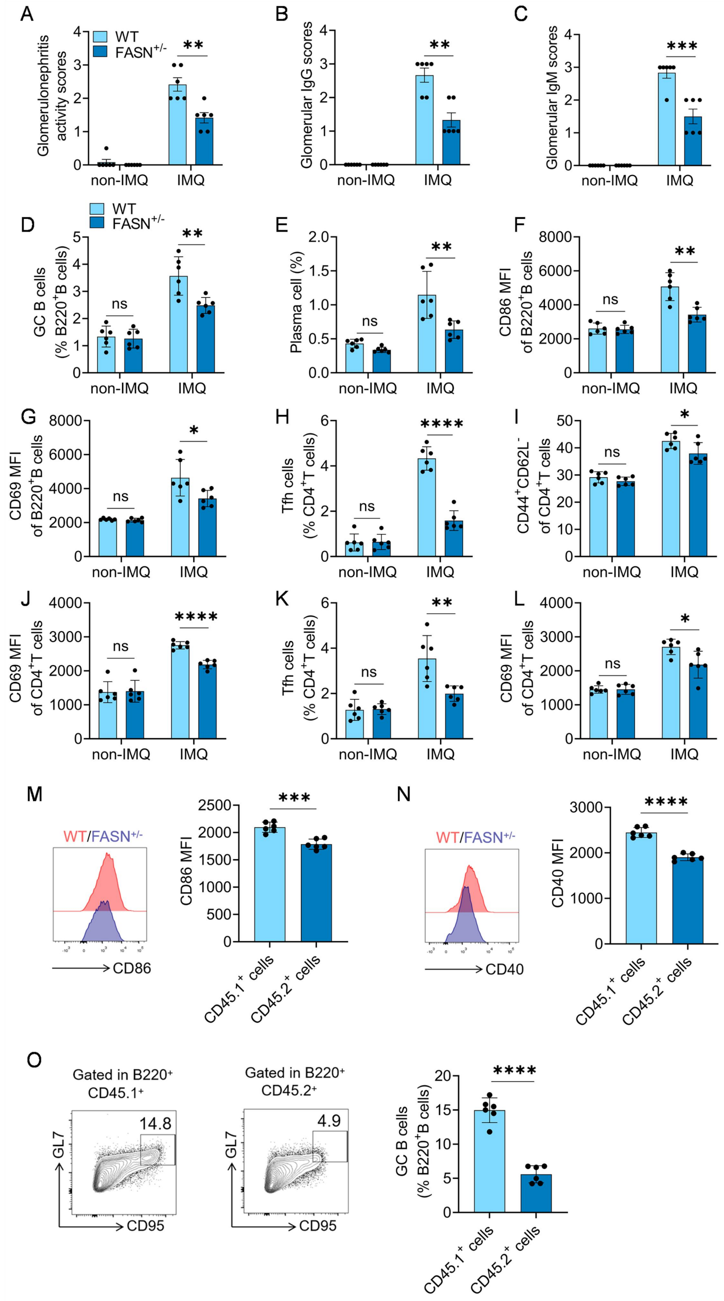

Supplemental Figure 12. Absence of FASN inhibits the activation and differentiation of B cells. (**A**) Glomerulonephritis activity scores, (**B**) glomerular IgG scores and (**C**) glomerular IgM scores in IMQ-treated wild-type or *FASN*$^{+/-}$ mice. The percentage of (**D**) GC B cells, (**E**) plasma cells and the expression of (**F**) CD86 and (**G**) CD69 on B220^{+} B cells in mLNs and the percentage of (**H**) Tfh cells, (**I**) memory CD4^{+} T cells and the expression of (**J**) CD69 on CD4^{+} T cells in spleen of IMQ-treated wild-type or *FASN*$^{+/-}$ mice. The percentage of (**K**) Tfh cells and the expression of (**L**) CD69 on CD4^{+} T cells in the mLNs of IMQ-treated wild-type or *FASN*$^{+/-}$ mice. The expression of (**M**) CD86 and (**N**) CD40 on the surface of CD45.1^{+} B cells and CD45.2^{+} B cells in the mLNs and the percentage of (**O**) GC B cells in CD45.1^{+} B cells and CD45.2^{+} B cells in the mLNs. The data are shown as the means ± SEM and are representative of three independent experiments (n=6 mice/group). *p <

0.05, **$p < 0.01$, ***$p < 0.001$, ****$p < 0.0001$, ns denotes $p > 0.05$, and as determined by two-tailed Student's *t*-test or two-way ANOVA.

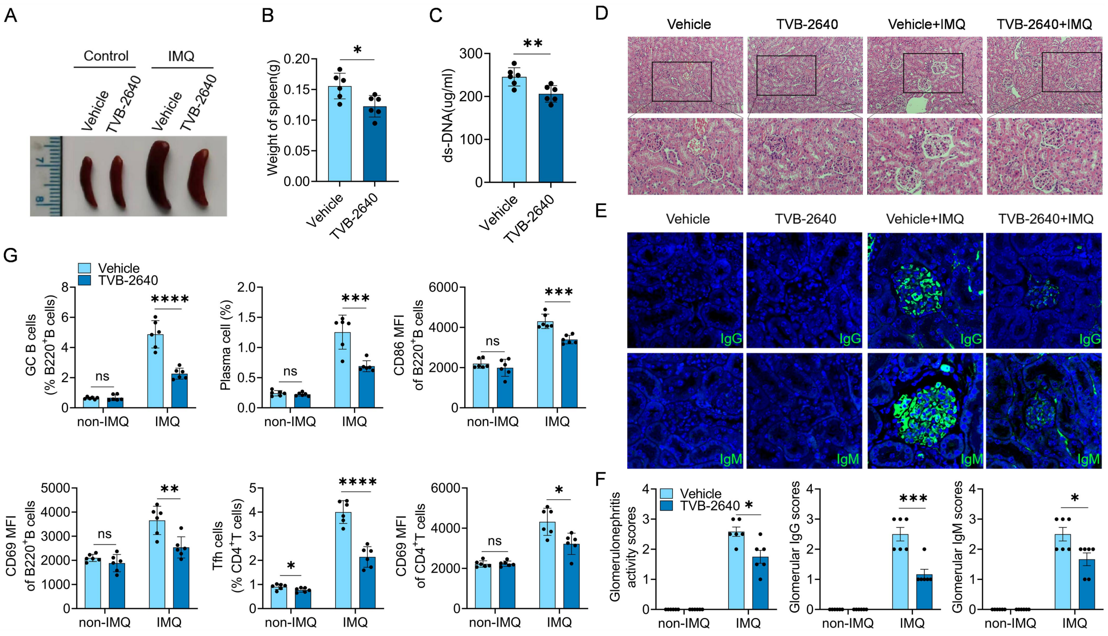

Supplemental Figure 13. TVB-2640 alleviates the progression of IMQ-induced lupus in mice. (**A**) Splenic images, (**B**) spleen weights, (**C**) serum levels of anti-dsDNA antibodies, (**D-F**) H&E staining of kidney, and renal IgG and IgM deposition in IMQ mice treated with vehicle or TVB-2640. (**G**) FACS analysis of the percentages of spleen GC B cells ($B220^+GL\text{-}7^+CD95^+$), plasma cells ($CD138^+B220^-$), the expression of CD86 and CD69 on $B220^+$ B cells and percentage of Tfh cells ($CD4^+CXCR5^+PD\text{-}1^+$) and the expression of CD69 on $CD4^+$ T cells in IMQ mice treated with vehicle or TVB-2640. The data are shown as the means ± SEM and are representative of three independent experiments (n=6 mice/group). *$p < 0.05$, **$p < 0.01$, ***$p < 0.001$, ****$p < 0.0001$, ns denotes $p > 0.05$, and as determined by two-tailed Student's *t*-test or two-way ANOVA.

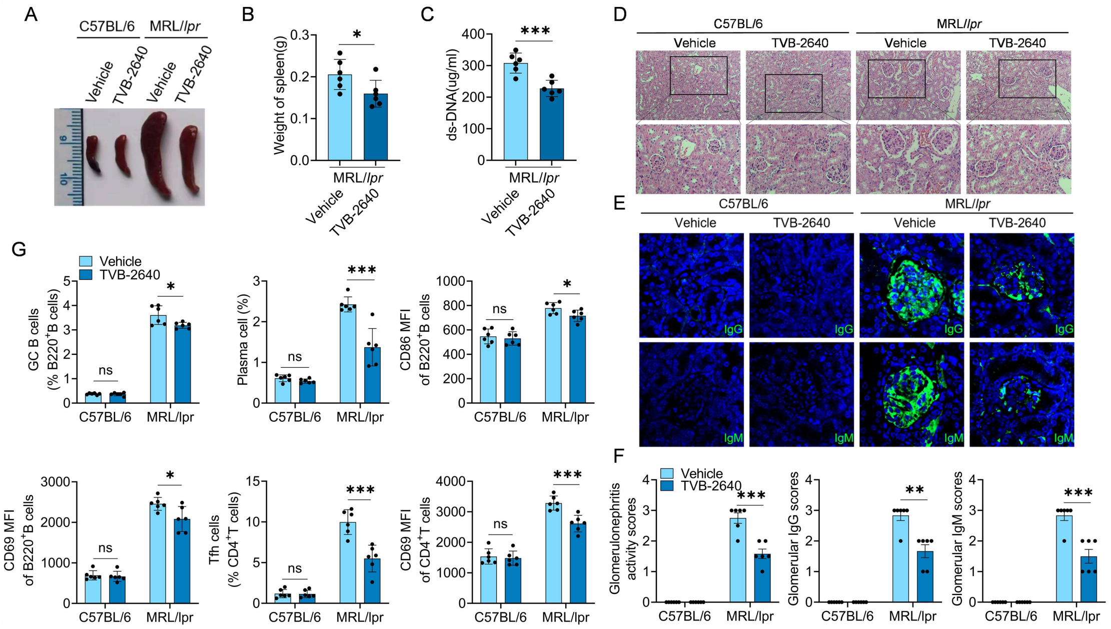

Supplemental Figure 14. TVB-2640 alleviates the progression of MRL/*lpr* lupus in mice. (**A**) Splenic images, (**B**) spleen weights, (**C**) serum levels of anti-dsDNA antibodies, (**D-F**) H&E staining of kidney, and renal IgG and IgM deposition in MRL/*lpr* mice treated with vehicle or TVB-2640. (**G**) FACS analysis of the percentages of spleen GC B cells, plasma cells, the expression of CD86 and CD69 on B220$^+$ B cells and percentage of Tfh cells and the expression of CD69 on CD4$^+$ T cells in MRL/*lpr* mice treated with vehicle or TVB-2640. The data are shown as the means ± SEM and are representative of three independent experiments (n=6 mice/group). **p* < 0.05, ***p* < 0.01, ****p* < 0.001, *****p* < 0.0001, ns denotes *p* > 0.05, and as determined by two-tailed Student's *t*-test or two-way ANOVA.

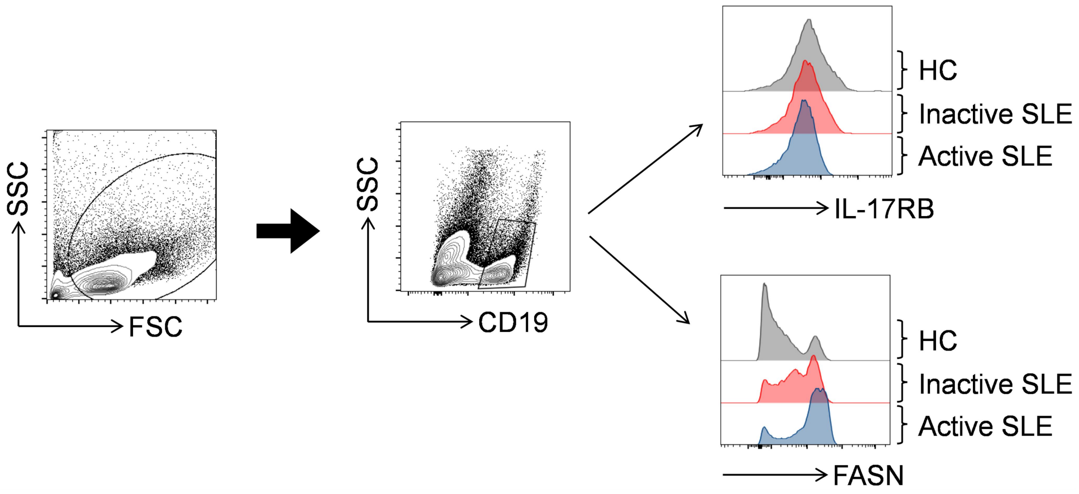

Supplemental Figure 15. Gating strategy for flow cytometry in Figure 6.

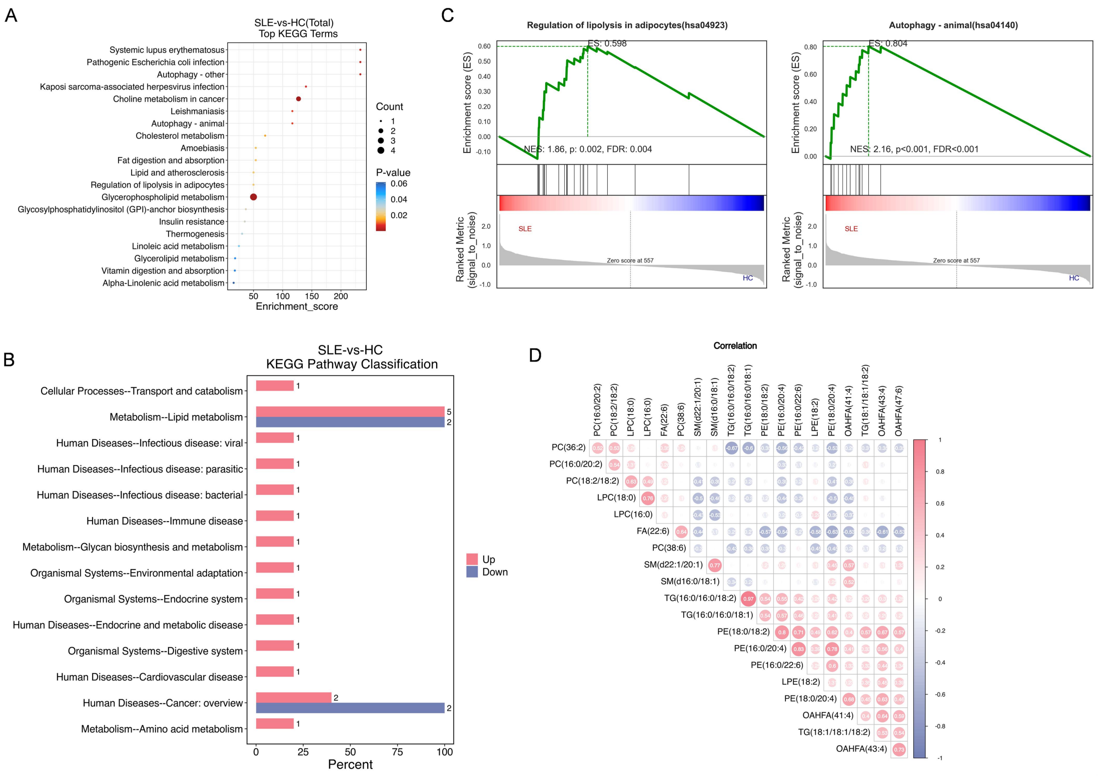

Supplemental Figure 16. Liposome profiling of healthy controls and SLE patients. (**A-B**) KEGG analysis, (**C**) GESA and (**D**) correlation analysis of metabolites generated from peripheral blood serum lipid profiles in healthy individuals and SLE patients.
