# Supplementary material for: IL-17B alleviates the pathogenesis of systemic lupus erythematosus by inhibiting FASN-mediated differentiation of B cells
Source: JCI Insight. 2024 Aug 8;9(18):e181906. doi: 10.1172/jci.insight.181906 (PMC11457847; doi:10.1172/jci.insight.181906)

Full unedited gel for Figure 3F

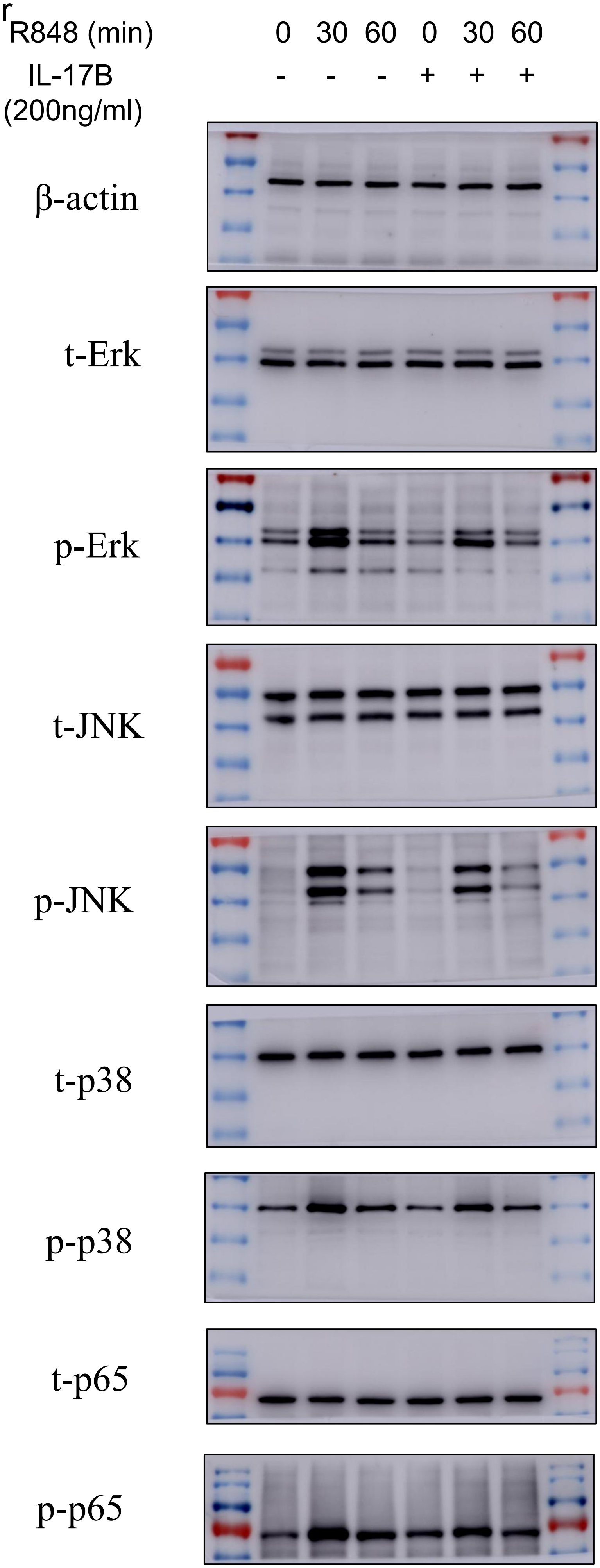

Full unedited gel for
Figure 4E

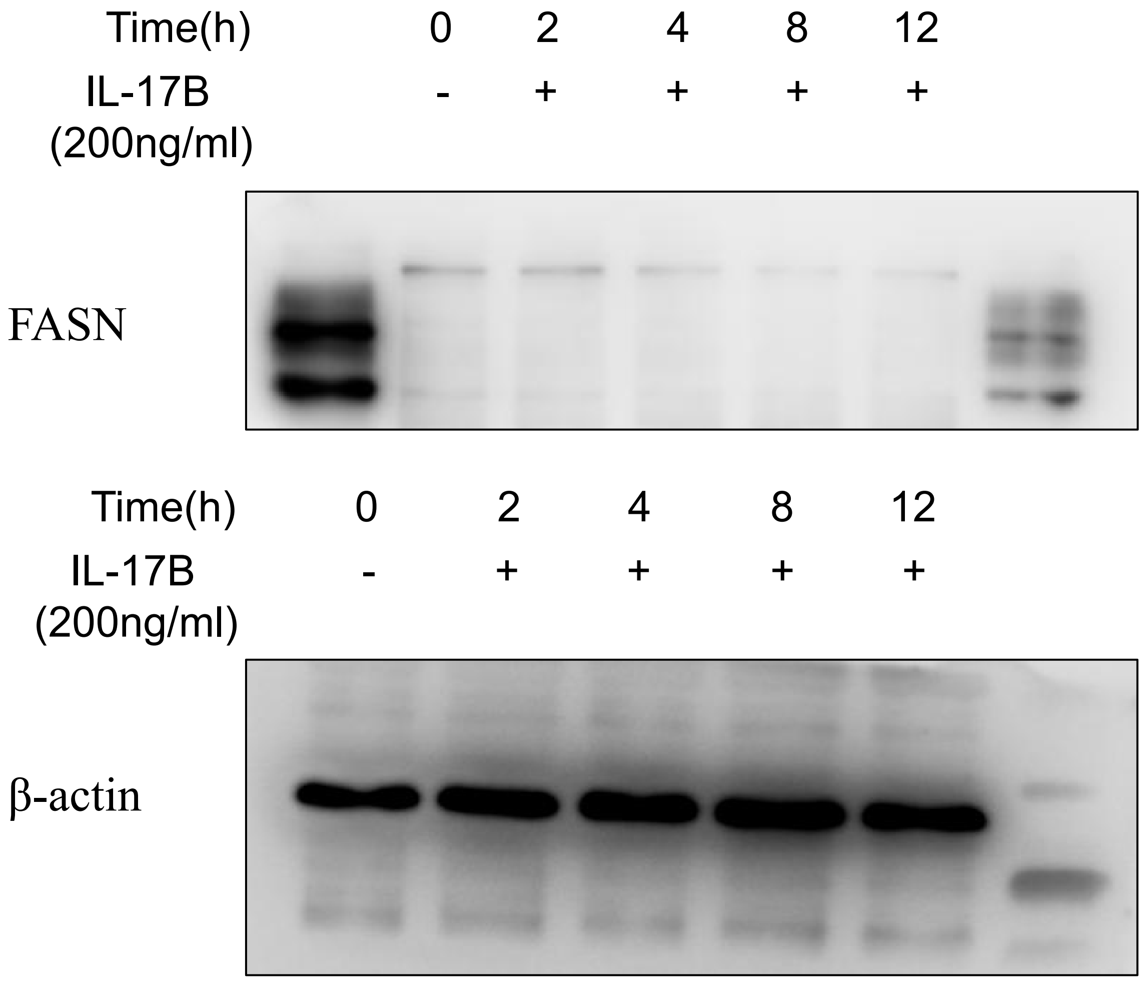

Full unedited gel for Figure 4M

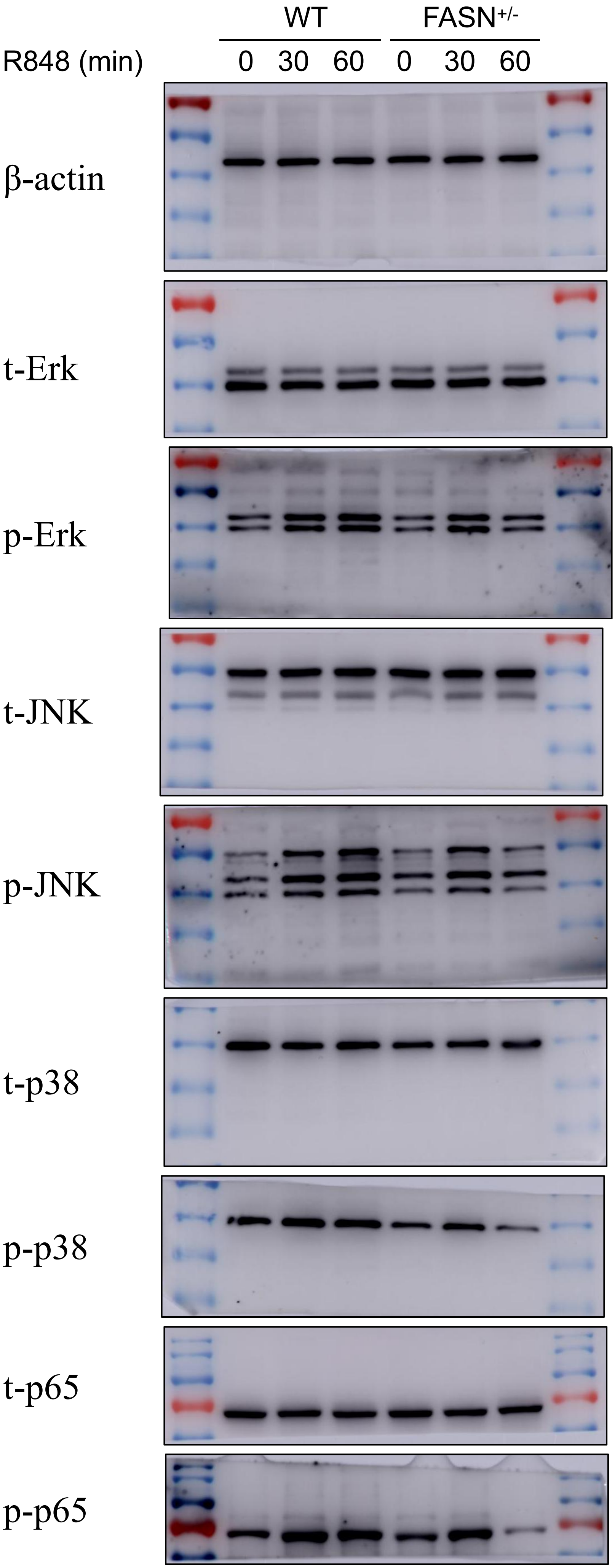

Full unedited gel for supplementary Figure 10D

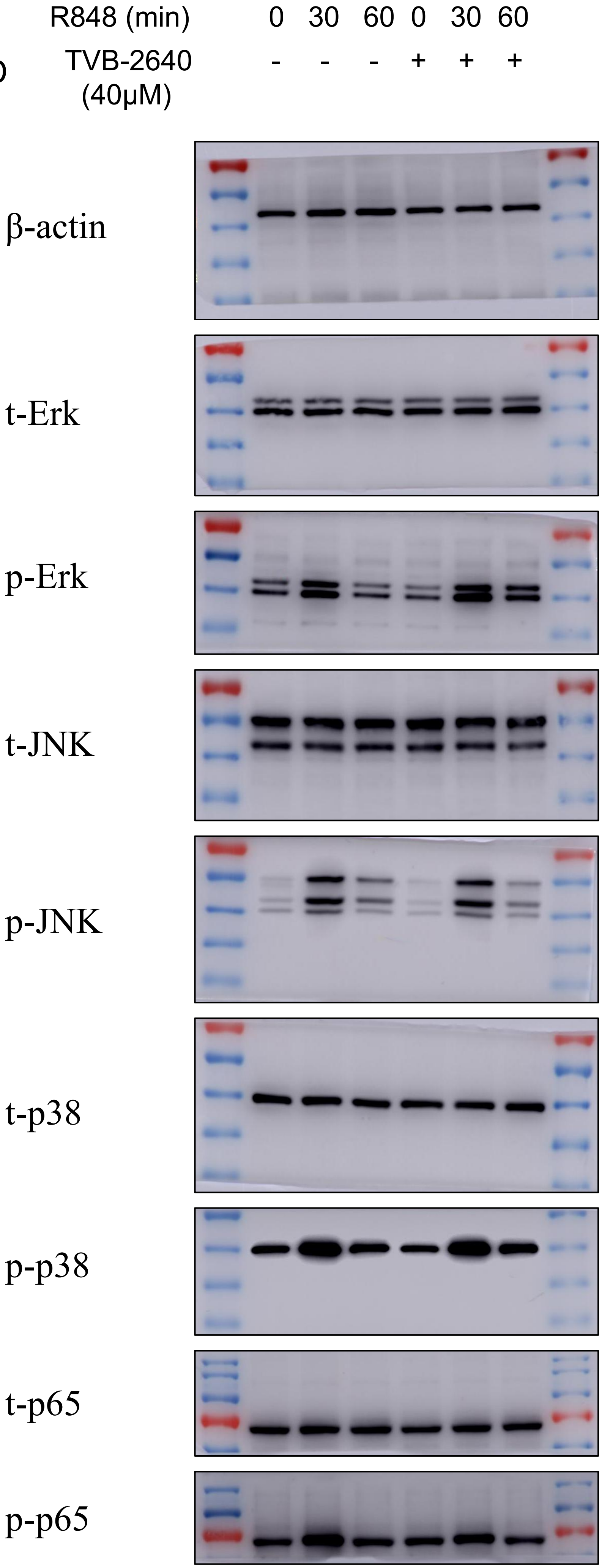

Full unedited gel for supplementary Figure 11D

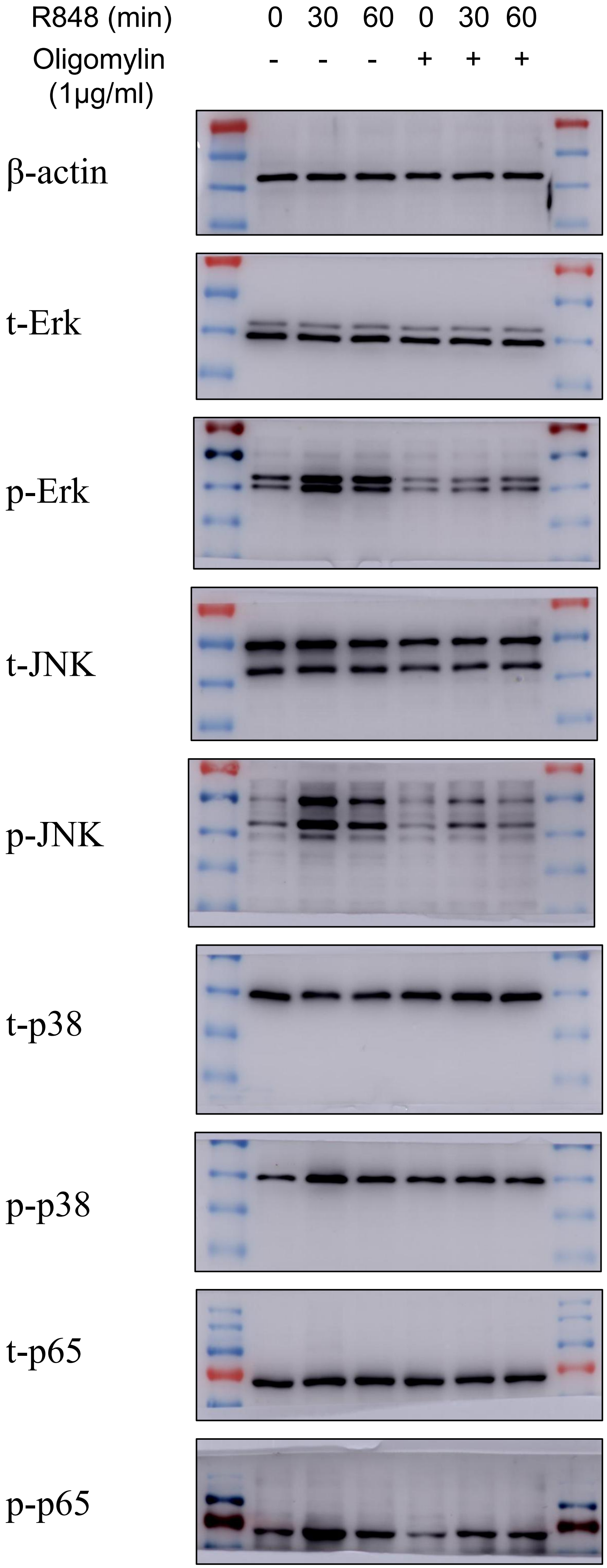

Supplement: Unedited blot and gel images [file jciinsight-9-181906-s092.pdf]
